# ORC6 marks a replication-active malignant epithelial state and is associated with immune-low features in oral squamous cell carcinoma

**DOI:** 10.3389/fonc.2026.1882429

**Published:** 2026-07-21

**Authors:** Shaofu Yan, Wenqi Tan, Yanxin Zhang, Jinyan Si, Jiehua Guo, Bo Yang, Jing Shi

**Affiliations:** 1 Shanxi Medical University School and Hospital of Stomatology, Taiyuan, China; 2 The Fifth Clinical Medical College of Shanxi Medical University, Taiyuan, China; 3 Shanxi Oral Health Prevention and Control Technology Innovation Center, Taiyuan, China

**Keywords:** antigen presentation, malignant epithelial cells, oral squamous cell carcinoma, ORC6, replication-active state, single-cell transcriptomics, tumor microenvironment

## Abstract

**Introduction:**

Oral squamous cell carcinoma (OSCC) shows marked biological heterogeneity, but markers that connect malignant epithelial states with the tumor immune context remain limited. Origin recognition complex subunit 6 (ORC6) participates in DNA replication licensing, but its disease-specific role in OSCC is not well defined.

**Methods:**

We integrated anatomically filtered bulk transcriptomic data, external validation cohorts, single-cell transcriptomic profiling, immune-context analyses, proliferation-adjusted sensitivity analyses, virtual perturbation, and in vitro ORC6-knockdown assays to evaluate the clinical and biological relevance of ORC6 in OSCC.

**Results:**

After reconstruction of a strictly defined TCGA oral cavity OSCC cohort excluding base of tongue and other non-oral cavity subsites, ORC6 remained upregulated in OSCC tissues and cell lines and was associated with adverse clinicopathological features and poorer survival. Exploratory analysis in the HPV-negative GSE41613 cohort further supported the adverse survival association of ORC6. Single-cell analysis showed preferential enrichment of ORC6 in malignant epithelial cells. Within this compartment, high ORC6 expression marked a replication-active state characterized by activation of DNA-replication and cell-cycle programs and weaker transcriptional signatures of interferon response and antigen presentation. These differences were directionally preserved in donor-matched pseudobulk comparisons and after cell-cycle-score regression. At the bulk-tumor level, proliferation-adjusted models showed that ORC6 retained directionally inverse associations with selected immune-related features, including cytolytic activity, IFN-γ response, and MHC-I/antigen-presentation signatures. ORC6 knockdown in CAL27 cells reduced cell growth, migration, invasion, and S-phase fraction while increasing antigen-presentation-associated transcripts and surface HLA-A/B/C expression.

**Discussion:**

These findings support ORC6 as a marker of a replication-active malignant epithelial state associated with attenuated immune-related features in OSCC, particularly antigen-presentation-related programs. Further functional immune-recognition studies and independent clinical validation are warranted.

## 1 Introduction

Oral squamous cell carcinoma (OSCC) is one of the most common and aggressive subtypes of head and neck squamous cell carcinoma (HNSCC). It is characterized by local invasion, high rates of recurrence and metastasis, and poor prognosis. According to the Global Cancer Observatory, there were an estimated 377,713 new cases and 177,757 deaths from lip and oral cavity cancer worldwide in 2020, and this burden is projected to increase further (1).

A multimodal treatment strategy centered on surgery is well established for HNSCC and OSCC; however, recurrent or metastatic disease remains difficult to manage in a biologically stratified manner (2). Immunotherapy, particularly PD-1 inhibitors, has gradually been integrated into the management of recurrent or metastatic HNSCC. Pivotal phase III trials, including CheckMate 141 and KEYNOTE-048, have established the role of PD-1 blockade in both second-line and first-line treatment settings (3, 4).

In recent years, the tumor microenvironment (TME) and tumor cell–intrinsic transcriptional programs have been increasingly recognized as key determinants of tumor progression, prognosis, and treatment response. Distinct immune infiltration patterns—such as inflamed, excluded, and cold phenotypes—are closely associated with clinical outcomes and therapeutic sensitivity (5, 6). However, many public studies continue to analyze OSCC within the anatomically heterogeneous framework of HNSCC, which may obscure OSCC-specific signals with biological and translational relevance (7).

In this study, we localized malignant epithelial cells and characterized their intrinsic transcriptional states at single-cell resolution. This analytical approach is supported by prior single-cell studies indicating that epithelial state heterogeneity, partial EMT programs, and stromal–immune coupling are major determinants of HNSCC and OSCC behavior (8, 9).

Origin recognition complex subunit 6 (ORC6) is a key component of the origin recognition complex (ORC). It participates in replication origin recognition, pre-replication complex assembly, and S-phase progression, and also contributes to replication fork stability and genome maintenance. Recent mechanistic studies have shown that ORC6 localizes to replication forks, and that its DNA-binding properties and regulated subcellular localization influence replication efficiency and genome surveillance (10, 11).

Pan-cancer analyses have demonstrated that ORC6 is upregulated in multiple solid tumors and is associated with aggressive biological behavior. In tumor models such as non-small cell lung cancer, ORC6 upregulation has been linked to more aggressive phenotypes (12, 13). However, research on ORC6 in HNSCC has largely been limited to cohort-based associations, and systematic evidence in OSCC as a distinct clinical entity remains sparse.

**Figure 1:**
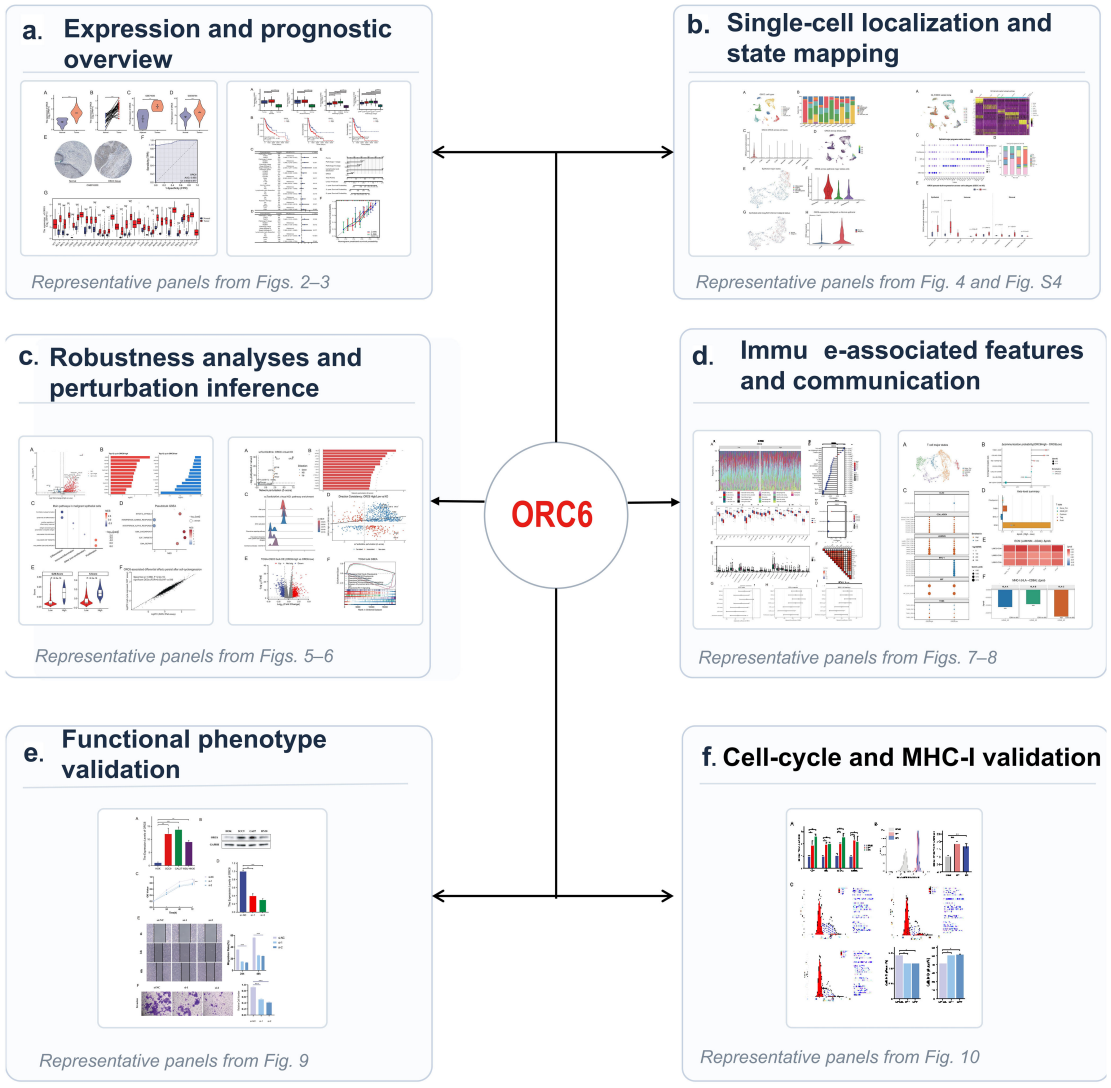
Integrated study overview. **(a)** Expression and prognostic overview. **(b)** Single-cell localization and state mapping. **(c)** Robustness analyses and perturbation inference. **(d)** Immune-associated features and cell–cell communication. **(e)** Functional phenotype validation. **(f)** Cell-cycle and MHC-I validation.

To address these clinical and biological gaps, we asked whether ORC6 identifies a replication-active malignant epithelial state in OSCC and whether this state remains associated with immune-low features after accounting for cell-cycle-related confounding. We therefore constructed an integrative analytical framework combining bulk transcriptomic analysis, independent expression validation, single-cell mapping, donor-paired pseudobulk validation, proliferation-adjusted immune analyses, virtual perturbation, and *in vitro* functional experiments. Our overall study design is summarized in **Figure 1**.

## 2 Materials and methods

### 2.1 Study design and data sources

This study comprised bulk transcriptomic analysis, clinicopathologic and survival analysis, single-cell transcriptomic analysis, immune-context analysis, proliferation-adjusted sensitivity analyses, and experimental validation. Public transcriptomic datasets were obtained from TCGA, GEO, GTEx, and GSE181919. To address anatomical heterogeneity within HNSCC, TCGA-based OSCC analyses were reconstructed using a strict oral cavity definition, with exclusion of base of tongue and other non-oral cavity subsites where applicable. Single-cell analyses were conducted in tumor-derived OSCC samples. Immune-related analyses were performed at both bulk and single-cell levels, with particular attention to proliferation-related confounding because ORC6 is intrinsically linked to DNA replication and cell-cycle progression. Experimental validation was performed at the cell level using ORC6 knockdown in CAL27 cells, including functional assays and flow cytometric assessment of surface HLA-A/B/C expression. Exploratory reversal-signature and structure-based analyses were moved to the Supplementary Materials and were interpreted only as hypothesis-generating information, not as evidence of direct ORC6 druggability, therapeutic efficacy, or clinical actionability.

### 2.2 Bulk transcriptomic data collection and preprocessing

Bulk RNA-seq and corresponding clinicopathologic data were obtained from TCGA. To construct a strictly defined TCGA-OSCC cohort, only primary tumor samples arising from oral cavity subsites were retained. Cases annotated as base of tongue or other non-oral cavity HNSCC subsites, including oropharyngeal, laryngeal, hypopharyngeal, and tonsillar tumors, were excluded. After strict anatomical filtering and retention of primary tumor samples, 306 TCGA-OSCC tumor samples were retained for expression-based and immune-context analyses when the required data were available. For OS-based survival analyses, patients were further required to have valid survival information, defined as non-missing OS time, OS time > 0, and non-missing OS status; this yielded 276 patients for OS, Kaplan-Meier, and Cox regression analyses. TCGA adjacent normal tissues were used only for tumor-normal expression and ROC analyses and were not included in clinicopathological or survival analyses. GEO microarray datasets used for external validation were downloaded from the Gene Expression Omnibus. When multiple probes mapped to the same gene symbol, probe values were collapsed at the gene level using the mean expression value across probes. GEO expression matrices were normalized using limma. Pan-cancer expression data were obtained from TCGA-GTEx resources for descriptive comparison only, whereas all OSCC-specific conclusions were based on anatomically filtered OSCC cohorts. Pan-cancer survival endpoint data were obtained from the curated TCGA clinical resource reported by Liu et al. (14).

### 2.3 Clinicopathologic association, diagnostic evaluation, and prognostic analysis

Associations between ORC6 expression and clinicopathologic variables were evaluated using statistical tests appropriate for variable type and distribution. Diagnostic performance was assessed by receiver operating characteristic analysis. Survival outcomes, including overall survival (OS), progression-free interval (PFI), and disease-specific survival (DSS), were evaluated using Kaplan–Meier analysis with the log-rank test. For OS, candidate clinicopathologic variables, including ORC6 group, pathologic T stage, pathologic N stage, pathologic stage, histologic grade, gender, age, alcohol history, lymph node neck dissection, smoking status, and radiation therapy, were first assessed by univariable Cox proportional hazards regression. Variables with P< 0.05 in the univariable analysis were then entered into the multivariable Cox regression model. A reduced prognostic nomogram for 1-, 3-, and 5-year OS prediction was constructed using predictors that remained statistically significant in the multivariable Cox model. Nomogram performance was evaluated using the concordance index (C-index) and calibration curves. All tests were two-sided, and P< 0.05 was considered statistically significant. An exploratory external survival validation was performed in the HPV-negative GSE41613 OSCC cohort using Kaplan-Meier analysis and univariable Cox regression.

#### 2.3.1 Stratification rules across analytical levels

ORC6 stratification was defined according to the analytical level. At the patient level in bulk cohorts, tumors were dichotomized by the cohort median. In malignant epithelial single-cell analyses, ORC6-high cells were defined by the upper quartile of ORC6 expression, and the remaining malignant epithelial cells were classified as ORC6-low. In donor-paired pseudobulk analyses, a within-sample rank-based 50/50 split was used to define ORC6-high and ORC6-low groups. These rules were selected to match the statistical structure of each analysis while preserving interpretability across levels.

### 2.4 Bulk differential expression, enrichment analysis, and feature prioritization

Differential expression analysis in bulk RNA-seq data was performed using DESeq2. Genes with FDR< 0.05 and |log2FC| > 1 were retained for downstream analyses. Functional enrichment analyses were performed using standard over-representation and gene set enrichment workflows. Machine-learning-assisted feature prioritization was performed using Boruta and SVM-RFE and was used to support candidate ranking rather than to build a predictive classifier.

### 2.5 Single-cell data preprocessing, integration, and annotation

OSCC single-cell RNA-sequencing data were obtained from GSE181919, and only 10 tumor tissue samples (tissue.type = “CA”; C04, C06, C07, C15, C21, C26, C30, C31, C51, and C60) were included. Single-cell analyses were performed in Seurat (v5.3.1). SCTransform was used for normalization and variance stabilization, with mitochondrial content (percent.mito) regressed out. Harmony (v1.2.4) was applied in PCA space using sample.id as the batch variable to generate integrated embeddings for downstream clustering and UMAP visualization (15, 16). Quality-control criteria were defined as follows: genes detected per cell ≥ 500, UMI counts ≥ 500, mitochondrial gene proportion ≤ 20%, and RNA complexity log10(nFeature_RNA)/log10(nCount_RNA) ≥ 0.65. After normalization, the top 2,000 highly variable genes were selected. A KNN graph was constructed in PCA space and Louvain clustering was performed, using a resolution of 0.5 for major clustering and 0.1 for lineage-level grouping. Initial cell annotation was performed using SingleR (v2.8.0) with the HPCA reference and was then refined on the basis of canonical markers and published literature. Epithelial cells and T cells were subsequently re-normalized, re-embedded, and re-clustered for subtype analysis.

### 2.6 Identification of malignant epithelial cells by CNV inference

Large-scale copy-number variation was inferred in epithelial cells using CopyKAT. Cells were classified as aneuploid or diploid and the predicted labels were written back to the Seurat metadata. All tumor-cell-intrinsic analyses were restricted to the CopyKAT-defined malignant epithelial compartment (17). CopyKAT was run with the following settings: id.type = “S”, ngene.chr = 5, win.size = 25, KS.cut = 0.1, distance = “euclidean”, norm.cell.names = NULL, and n.cores = 1.

### 2.7 ORC6 stratification in malignant epithelial cells and differential/enrichment analyses

Within CopyKAT-defined malignant epithelial cells, ORC6-high was defined using the upper quartile of ORC6 expression and the remaining cells were classified as ORC6-low (18). Differential expression between the two groups was performed using Seurat FindMarkers with the Wilcoxon rank-sum test. Significance thresholds were set at Benjamini–Hochberg-adjusted false discovery rate (FDR)< 0.05 and |log2FC| > 0.5. Ranked differential signals were used for downstream pathway analysis. Gene set enrichment analysis was performed on GO Biological Process and KEGG collections, and representative pathways were summarized across proliferation/replication, differentiation, immune/inflammation, and other biologically relevant categories.

#### 2.7.1 Donor-paired pseudobulk validation

To reduce pseudoreplication at the single-cell level, donor-aggregated paired pseudobulk analysis was performed. Raw UMI counts were extracted from the RNA assay counts layer. Within each sample, gene-level counts were summed across cells after ORC6-based stratification to generate sample-by-group pseudobulk profiles. Because some samples contained relatively few malignant epithelial cells, a within-sample rank-based 50/50 split was used for pseudobulk grouping. When ties occurred during within-sample ranking, they were resolved using a fixed random seed (set.seed (1)). Only samples with at least 30 cells per group were retained. Differential expression of pseudobulk profiles was analyzed using edgeR with TMM normalization and a paired design (~ donor + group) under the quasi-likelihood framework. At the pathway level, fgsea (v1.32.4) was performed using MSigDB Hallmark gene sets ranked by pseudobulk log2FC.

### 2.8 Control of cell-cycle confounding and sensitivity analyses

Because ORC6 is linked to DNA replication and cell-cycle regulation, cell-cycle correction was treated as a sensitivity analysis. S.Score and G2M.Score were calculated for each malignant epithelial cell using Seurat cell-cycle gene sets. These scores, together with mitochondrial proportion when available, were included as regression variables within the SCTransform framework to generate a cell-cycle-adjusted residual expression space. The ORC6 stratification and differential-analysis workflow was then repeated in the adjusted data. Concordance between pre- and post-adjustment differential effects was quantified using effect-size correlations and directional consistency rates. Regression efficacy was further evaluated by comparing gene-level correlations with S and G2M scores before and after adjustment.

### 2.9 Virtual knockout and network perturbation analysis

Virtual knockout of ORC6 was performed in malignant epithelial cells using scTenifoldKnk (v1.0.1). The malignant epithelial expression matrix was used to construct the single-cell gene regulatory network, after which in silico ORC6 perturbation was modeled according to the study workflow. Knockout effects were quantified using network perturbation Z scores and adjusted P values. The resulting perturbation signature was subjected to KEGG and Reactome enrichment analyses. To assess directional concordance, perturbed genes were compared with differential-expression-derived effect directions from the ORC6-high versus ORC6-low contrast.

### 2.10 Bulk immune deconvolution and proliferation-adjusted sensitivity analyses

To evaluate the relationship between ORC6 expression and the tumor immune context at the patient level, bulk immune analyses were performed in the strictly defined TCGA-OSCC cohort. ESTIMATE was used to infer ImmuneScore, StromalScore, and ESTIMATEScore. CIBERSORT was applied to estimate the relative abundance of immune cell subsets. In addition, ssGSEA/GSVA-based immune signature scoring was performed to quantify immune-related functional programs, including cytolytic activity, IFN-γ response, MHC-I/antigen-presentation signature, immune checkpoint-related features, and other immune programs of interest. GSVA was implemented using the GSVA package. Correlations between ORC6 expression and immune-related features were primarily assessed using Spearman correlation analysis.

Because ORC6 is intrinsically involved in DNA replication licensing and cell-cycle progression, proliferation-related confounding was explicitly considered. We first compared ORC6 expression with canonical proliferation markers and proliferation-related gene signatures, including MKI67, PCNA, TOP2A, MCM score, E2F score, G2/M score, and a proliferation metagene score. Single-gene markers were analyzed using normalized expression values. Multigene scores were calculated as the mean z-scored expression of genes within each predefined gene set. Detailed gene lists and score-construction procedures are provided in the Supplementary Methods.

To assess whether ORC6-associated immune relationships remained detectable after accounting for canonical proliferation programs, multivariable linear regression models were fitted for representative immune outcomes. The immune outcomes included cytolytic activity score, IFN-γ response score, and MHC-I/antigen-presentation score. Each proliferation-related covariate was incorporated separately into the model to avoid excessive collinearity among proliferation variables. The general model form was:


immune outcome ~ ORC6 expression + proliferation covariate

where ORC6 expression was modeled as a continuous variable and the proliferation covariate represented MKI67, PCNA, TOP2A, MCM score, E2F score, G2/M score, or the proliferation metagene score. The adjusted regression coefficient of ORC6 was extracted from each model and visualized to compare the direction and robustness of ORC6-associated immune relationships across different proliferation-adjustment strategies.

To reduce the influence of extreme values, selected proliferation-adjusted immune models were repeated after winsorization of ORC6 expression and/or immune-module values at the upper and lower 1% of the distribution. Directional consistency of regression coefficients before and after winsorization was then evaluated.

Clinical- and proliferation-adjusted survival sensitivity analyses were also performed to assess whether the adverse survival association of ORC6 was influenced by canonical proliferation programs after accounting for clinicopathologic covariates. Separate Cox proportional hazards models were fitted for overall survival, with each model incorporating ORC6 group, pathologic T stage, pathologic N stage, age, lymph node neck dissection, and one proliferation-related covariate. ORC6 was analyzed using median-based high versus low stratification, with the ORC6-low group used as the reference. The general model form was:


Surv(OS time, OS status) ~ ORC6 group + pathologic T stage + pathologic N stage + age + lymph node neck dissection + proliferation covariate

where the proliferation covariate represented MKI67, PCNA, TOP2A, MCM score, E2F score, G2/M score, or the proliferation metagene score. Cox models were fitted using complete cases for all variables included in each model. Hazard ratios and 95% confidence intervals for ORC6-high versus ORC6-low tumors were extracted from each model. These proliferation-adjusted analyses were interpreted as sensitivity analyses rather than as definitive evidence of proliferation-independent prognostic or immune-regulatory effects. Given the established biological link between ORC6 and DNA replication/cell-cycle progression, these analyses were used to determine whether ORC6-associated immune and survival patterns remained directionally consistent after accounting for canonical proliferation-related covariates.

### 2.11 Cell–cell communication analysis

Cell–cell communication was evaluated at single-cell resolution using CellChat (v1.6.1). Malignant epithelial cells jointly defined by CopyKAT and manual annotation were stratified by ORC6 expression, and communication from malignant epithelial cells to T-cell states was inferred under ORC6-high and ORC6-low conditions. Independent CellChat objects were constructed for the two conditions using the curated ligand–receptor knowledge base, and communication probability was estimated at both the interaction-pair and pathway levels. Low-abundance communication events were filtered by requiring a minimum of 20 participating cells (min.cells = 20).

#### 2.11.1 Quantification of differential communication

Differences in communication strength were quantified as:


Δprob = prob(ORC6High) − prob(ORC6Low)

where Δprob > 0 indicated relative enhancement under the ORC6-high condition and Δprob< 0 indicated relative attenuation. Differential interaction pairs were summarized at both the pathway and axis levels, and representative ligand–receptor examples were selected for interpretation. Ligand and receptor expression summaries were also examined to support biological interpretability.

#### 2.11.2 Interpretive boundaries

CellChat analysis was used as an inference framework based on transcript abundance and curated ligand–receptor priors. It was used to generate testable interaction hypotheses rather than to establish direct intercellular signaling changes *in vivo*.

### 2.12 Experimental validation

#### 2.12.1 Cell culture and siRNA transfection

Human OSCC cell lines CAL27, WSU-HN30, and SCC9, together with normal human oral keratinocytes (HOK), were purchased from Zhejiang Beidi Biotechnology Co., Ltd. Cell line identities were verified by short tandem repeat (STR) profiling, and routine mycoplasma testing was negative. Cells were cultured in complete medium supplemented with 10% fetal bovine serum and 1% penicillin–streptomycin at 37 °C in 5% CO_2_.

For loss-of-function experiments, CAL27 cells were transfected with two independent ORC6-targeting siRNAs (siORC6–1 and siORC6-2) or a non-targeting control siRNA (siNC) using Lipofectamine 2000 according to the manufacturer’s instructions. Cells were seeded 24 h before transfection to reach approximately 40%–60% confluence at the time of transfection.

#### 2.12.2 Quantitative real-time PCR

Total RNA was extracted using a commercial RNA extraction kit, followed by reverse transcription and quantitative real-time PCR according to the manufacturers’ protocols. Relative mRNA expression levels were calculated using the 2^-ΔΔCt method, with GAPDH as the internal reference.

To preliminarily assess whether ORC6 knockdown was accompanied by changes in tumor cell-intrinsic immune-related features, the expression levels of selected antigen-presentation-associated genes, including TAP1, B2M, HLA-A, and NLRC5, were additionally measured in CAL27 cells after siRNA transfection. Primer sequences are listed in the Supplementary Materials **Supplementary Table 1**.

#### 2.12.3 Western blot analysis

Total protein was extracted using RIPA lysis buffer supplemented with 1% PMSF. Protein samples were separated by 12% SDS-PAGE and transferred onto PVDF membranes. After blocking, membranes were incubated with primary antibodies against ORC6 and GAPDH, followed by the corresponding HRP-conjugated secondary antibodies. Protein bands were visualized using an ECL detection system.

Western blotting was used to compare basal ORC6 protein expression across HOK, SCC9, CAL27, and WSU-HN30 cells.

#### 2.12.4 Cell proliferation, migration, invasion, and cell-cycle assays

Cell proliferation/viability was assessed using the CCK-8 assay. Cells were seeded into 96-well plates, and OD450 values were measured at the indicated time points. Each condition included at least five replicate wells, and all experiments were independently repeated at least three times.

Cell migration was evaluated using the wound-healing assay. After transfection, cells were cultured to near confluence and a linear wound was generated using a sterile pipette tip. Cells were then maintained under low-serum conditions, and images were acquired at 0 h, 24 h, and 48 h at matched positions. Wound closure was quantified using ImageJ.

Cell invasion was assessed using 8-μm Transwell chambers. For invasion experiments, the upper chambers were precoated with Matrigel. After incubation, invading cells on the lower membrane surface were fixed, stained with crystal violet, and counted in at least five randomly selected microscopic fields. Each condition included triplicate wells, and all experiments were independently repeated at least three times.

For cell-cycle analysis, cells were fixed in 70% ice-cold ethanol, treated with RNase A, and stained with propidium iodide (PI) in the dark. DNA content was analyzed by flow cytometry, and cell-cycle distribution was fitted using FlowJo, yielding the proportions of cells in G1 and S phases.

#### 2.12.5 Flow cytometric analysis of surface HLA-A/B/C expression

Surface HLA-A/B/C expression was assessed by flow cytometry in CAL27 cells after siRNA transfection. Briefly, cells were harvested 48 h after transfection, washed with cold PBS, and stained with PE-conjugated mouse anti-human HLA-A/B/C monoclonal antibody (clone W6/32; BD Biosciences) for 30 min at 4 °C in the dark. Unstained and isotype controls were used for gating and background assessment. Data were acquired using a BD FACSCanto™ II flow cytometer and analyzed with FlowJo v10 software. After gating on intact single cells, surface HLA-A/B/C expression was quantified by mean fluorescence intensity (MFI) and normalized to the siNC group. Three independent biological replicates were performed.

#### 2.12.6 Statistical analysis for cell experiments

Data are presented as mean ± SD unless otherwise specified. For comparisons among three groups, one-way ANOVA followed by appropriate *post hoc* multiple-comparison testing was used. For experiments involving both group and time effects, two-way ANOVA was used. All tests were two-sided, and P< 0.05 was considered statistically significant.

### 2.13 Exploratory reversal-signature and structure-based compound prioritization

Exploratory reversal-signature and structure-based compound-prioritization analyses were moved to the Supplementary Materials. These analyses were performed only to generate testable hypotheses related to the ORC6-high transcriptional state and were not used to support direct ORC6 binding, ORC6 druggability, therapeutic efficacy, or clinical actionability. Details of reversal-guided compound screening, ORC6 structure preparation, pocket prediction, molecular docking, rescoring, and ADMET evaluation are provided in the Supplementary Methods.

### 2.14 Statistical analysis and software environment

All statistical analyses were performed in R unless otherwise specified. Multiple testing was controlled using the Benjamini–Hochberg false discovery rate procedure. Correlation analyses were performed primarily using Spearman’s method. Group comparisons were conducted using parametric or nonparametric tests according to data distribution and variance characteristics. Unless otherwise specified, all tests were two-sided and P< 0.05 was considered statistically significant. Confirmed packages and tools in the current workflow included Seurat (v5.3.1), harmony (v1.2.4), SingleR (v2.8.0), fgsea (v1.32.4), GSVA (v2.4.6), CellChat (v1.6.1), scTenifoldKnk (v1.0.1), and AutoDock Vina (v1.2.3), together with DESeq2, edgeR, ESTIMATE, CIBERSORT, and CopyKAT.

## 3 Results

### 3.1 ORC6 is upregulated in OSCC and shows cross-cohort consistency

**Figure 2:**
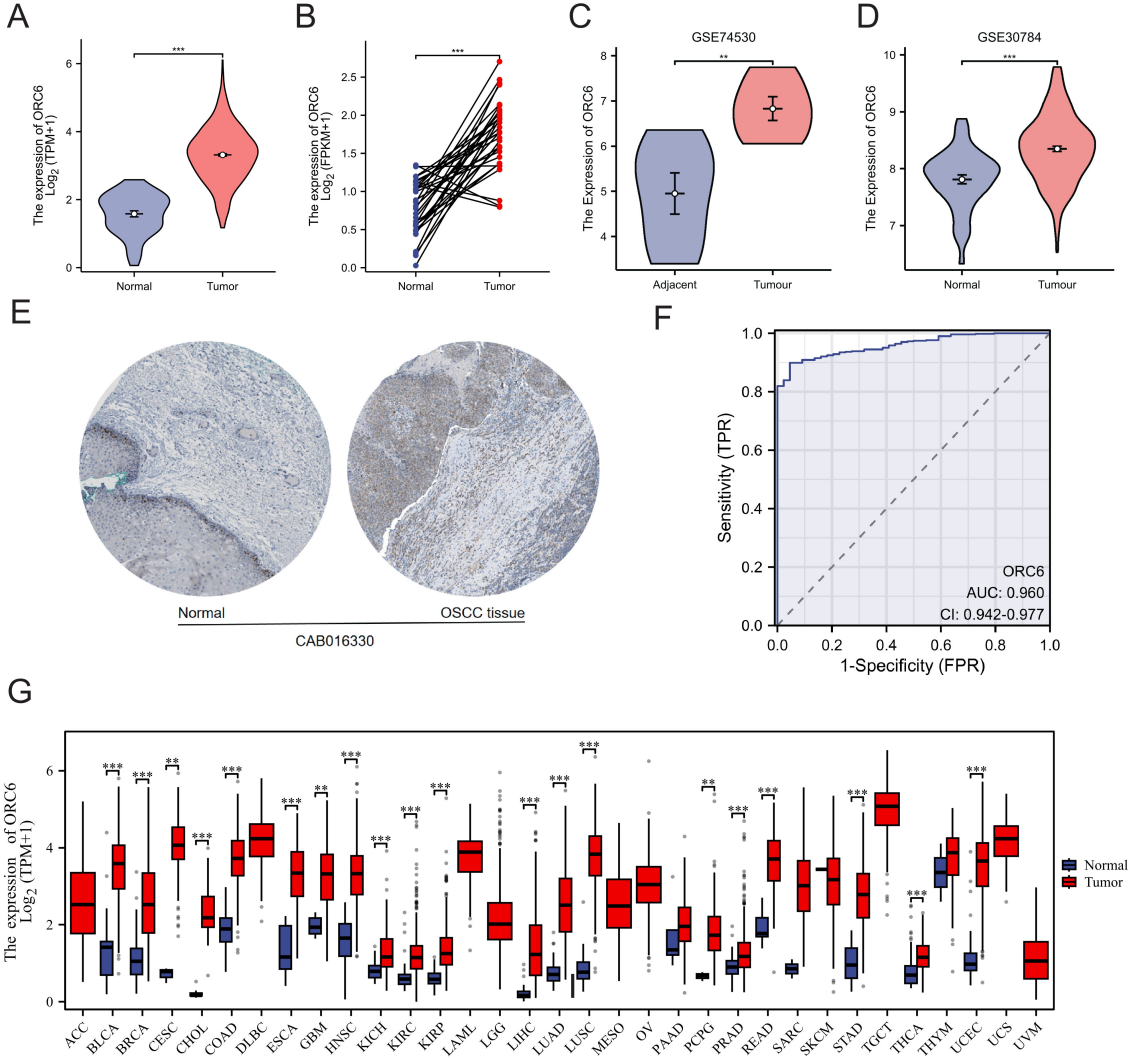
ORC6 is upregulated in OSCC and shows diagnostic potential. **(A)** ORC6 expression in tumor and normal tissues in the TCGA-OSCC cohort. **(B)** Comparison of ORC6 expression between OSCC tumors and matched normal tissues in the TCGA cohort. **(C)** Validation of differential ORC6 expression between tumor and adjacent non-tumor tissues in the GSE74530 dataset. **(D)** Validation of differential ORC6 expression between tumor and normal tissues in the GSE30784 dataset. **(E)** Representative immunohistochemical staining images of ORC6 in normal oral epithelium and OSCC tissues from the Human Protein Atlas. **(F)** Receiver operating characteristic (ROC) curve evaluating the performance of ORC6 in distinguishing tumor from normal tissues in the TCGA-OSCC cohort. **(G)** Pan-cancer overview of differential ORC6 expression across cancer types in the TCGA-GTEx dataset. #*P < 0.05, **P < 0.01, and ***P < 0.001.

We first evaluated ORC6 expression in bulk OSCC datasets. In the TCGA-OSCC cohort, ORC6 expression was significantly higher in tumor tissues than in normal tissues, and this difference remained evident in paired tumor-normal comparisons (**Figures 2A,B**). External validation in independent GEO cohorts showed the same direction of change: ORC6 was upregulated in tumors relative to adjacent non-tumor tissues in GSE74530 and relative to normal tissues in GSE30784 (**Figures 2C,D**). At the protein level, representative IHC images from the Human Protein Atlas (19) demonstrated elevated ORC6 expression in OSCC compared with normal oral mucosa (**Figure 2E**). In the TCGA-OSCC cohort, ROC analysis showed that ORC6 had strong discriminatory performance for distinguishing tumor from normal tissues, with an AUC of 0.960 (**Figure 2F**). A pan-cancer overview based on the TCGA-GTEx resource further showed that ORC6 was differentially expressed across multiple cancer types, with particularly prominent upregulation in several epithelial malignancies, including head and neck squamous cell carcinoma (**Figure 2G**). Supporting data including two machine learning approaches and pan-cancer prognostic analysis are provided in **Supplementary Figure 1**.

### 3.2 ORC6 is associated with adverse clinicopathologic features and poorer outcome in TCGA-OSCC

**Figure 3:**
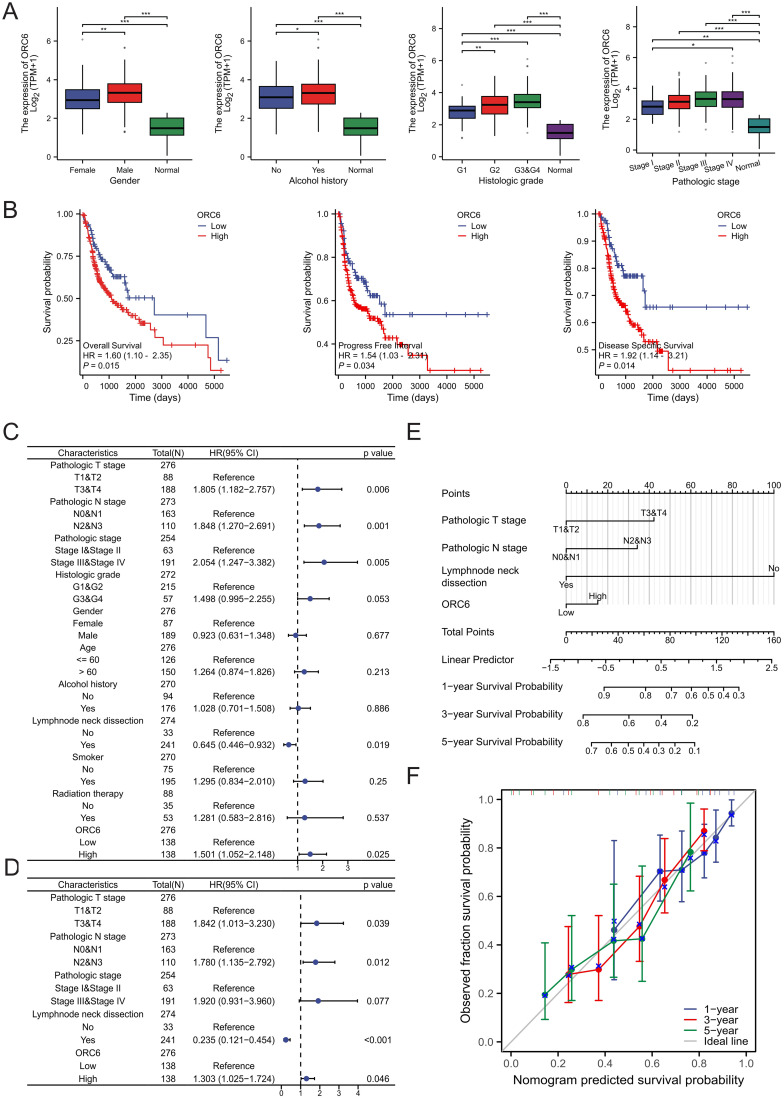
ORC6 is associated with clinicopathologic features and prognosis in TCGA-OSCC. **(A)** Associations between ORC6 expression and clinicopathologic variables. **(B)** Kaplan-Meier curves for overall survival (OS), progression-free interval (PFI), and disease-specific survival (DSS) in ORC6-high and ORC6-low tumors. **(C)** Univariate Cox regression analysis. **(D)** Multivariable Cox regression analysis. **(E)** Nomogram integrating ORC6 and clinicopathologic factors for 1-, 3-, and 5-year survival prediction.The nomogram achieved a C-index of 0.715 (95% CI, 0.691–0.739). **(F)** Calibration curves for the nomogram.Variables with P< 0.05 in univariable Cox regression were entered into the multivariable Cox model. The nomogram was then constructed using variables that remained statistically significant in the multivariable model. *P < 0.05, **P < 0.01, and ***P < 0.001.

After strict anatomical filtering and exclusion of base of tongue and other non-oral cavity HNSCC subsites, 306 primary TCGA-OSCC tumor samples were retained for expression-based analyses. For OS-based survival and Cox regression analyses, 276 patients with valid OS time and OS status were included. After median-based ORC6 stratification, the ORC6-high group showed less favorable clinicopathologic associations, particularly with respect to pathologic stage and histologic grade (**Figure 3A**; **Table 1**). Kaplan-Meier analysis showed that high ORC6 expression was associated with poorer OS, PFI, and DSS in the strictly defined TCGA-OSCC cohort (**Figure 3B**).

**Table 1:** Clinicopathologic characteristics according to ORC6 expression in the TCGA-OSCC cohort.

Characteristic	Low ORC6 expression	High ORC6 expression	P value
n	153	153	
Pathologic T stage, n (%)			0.021
T1	10 (6.5)	7 (4.6)	
T2	58 (37.9)	44 (28.8)	
T3	36 (23.5)	40 (26.1)	
T4	49 (32.0)	62 (40.5)	
Pathologic N stage, n (%)			0.121
N0	84 (55.6)	81 (53.3)	
N1	34 (22.5)	24 (15.8)	
N2-N3	33 (21.9)	47 (30.9)	
Pathologic stage, n (%)			0.045
Stage I	13 (9.4)	4 (2.8)	
Stage II	29 (21.0)	23 (15.9)	
Stage III	24 (17.4)	31 (21.4)	
Stage IV	72 (52.2)	87 (60.0)	
Gender, n (%)			0.015
Female	60 (39.2)	39 (25.5)	
Male	93 (60.8)	114 (74.5)	
Alcohol history, n (%)			0.031
No	59 (39.1)	42 (28.4)	
Yes	92 (60.9)	106 (71.6)	
Age, n (%)			0.277
<= 60	67 (43.8)	77 (50.7)	
> 60	86 (56.2)	75 (49.3)	
Lymphovascular invasion, n (%)			0.104
No	82 (73.9)	77 (65.3)	
Yes	29 (26.1)	41 (34.7)	
Anatomic neoplasm subdivision, n (%)			0.056
Alveolar Ridge	9 (5.9)	9 (5.9)	
Buccal Mucosa	8 (5.2)	14 (9.2)	
Floor of mouth	22 (14.4)	38 (24.8)	
Hard Palate	5 (3.3)	2 (1.3)	
Oral Cavity	40 (26.1)	32 (20.9)	
Oral Tongue	69 (45.1)	58 (37.9)	
Radiation therapy, n (%)			0.465
No	22 (44.0)	21 (35.6)	
Yes	28 (56.0)	38 (64.4)	

Percentages were calculated using the number of non-missing cases for each variable as the denominator. Missing or unknown values were excluded from percentage calculations and statistical comparisons.

In univariable Cox regression for OS, ORC6 was associated with poorer outcome (HR = 1.501, 95% CI 1.052-2.148, P = 0.025). Pathologic T stage (HR = 1.805, 95% CI 1.182-2.757, P = 0.006), pathologic N stage (HR = 1.848, 95% CI 1.270-2.691, P = 0.001), pathologic stage (HR = 2.054, 95% CI 1.247-3.382, P = 0.005), and lymph node neck dissection (HR = 0.645, 95% CI 0.446-0.932, P = 0.019) were also significantly associated with OS (**Figure 3C**). These variables were therefore entered into the multivariable Cox model.

In the multivariable Cox model, ORC6 remained modestly associated with poorer OS (HR = 1.303, 95% CI 1.025-1.724, P = 0.046), together with pathologic T stage (HR = 1.842, 95% CI 1.013-3.230, P = 0.039), pathologic N stage (HR = 1.780, 95% CI 1.135-2.792, P = 0.012), and lymph node neck dissection (HR = 0.235, 95% CI 0.121-0.454, P< 0.001). Pathologic stage was included in the multivariable model because it was significant in univariable analysis, but it did not remain statistically significant after adjustment (HR = 1.920, 95% CI 0.931-3.960, P = 0.077; **Figure 3D**). A reduced prognostic nomogram was therefore constructed using predictors that remained statistically significant in the multivariable model, including pathologic T stage, pathologic N stage, lymph node neck dissection, and ORC6 (**Figure 3E**). The nomogram achieved a C-index of 0.715 (95% CI, 0.691–0.739), indicating moderate discriminative performance in this retrospective TCGA-OSCC cohort. Calibration curves showed acceptable agreement between predicted and observed 1-, 3-, and 5-year OS probabilities (**Figure 3F**). Because these analyses were based on a retrospective public cohort, the prognostic value of ORC6 and the nomogram should be interpreted cautiously and require further validation in independent clinically annotated OSCC cohorts.

In the external GSE41613 OSCC cohort, high ORC6 expression was also associated with poorer OS (log-rank P = 0.0315; **Supplementary Figure 2A**). Univariable Cox analysis further supported the adverse survival association of ORC6 in this cohort (HR = 1.879, 95% CI 1.048-3.369, P = 0.034; **Supplementary Figure 2B**), providing exploratory support in an HPV-negative OSCC validation dataset.

### 3.3 ORC6 is preferentially localized to malignant epithelial cells at single-cell resolution

**Figure 4:**
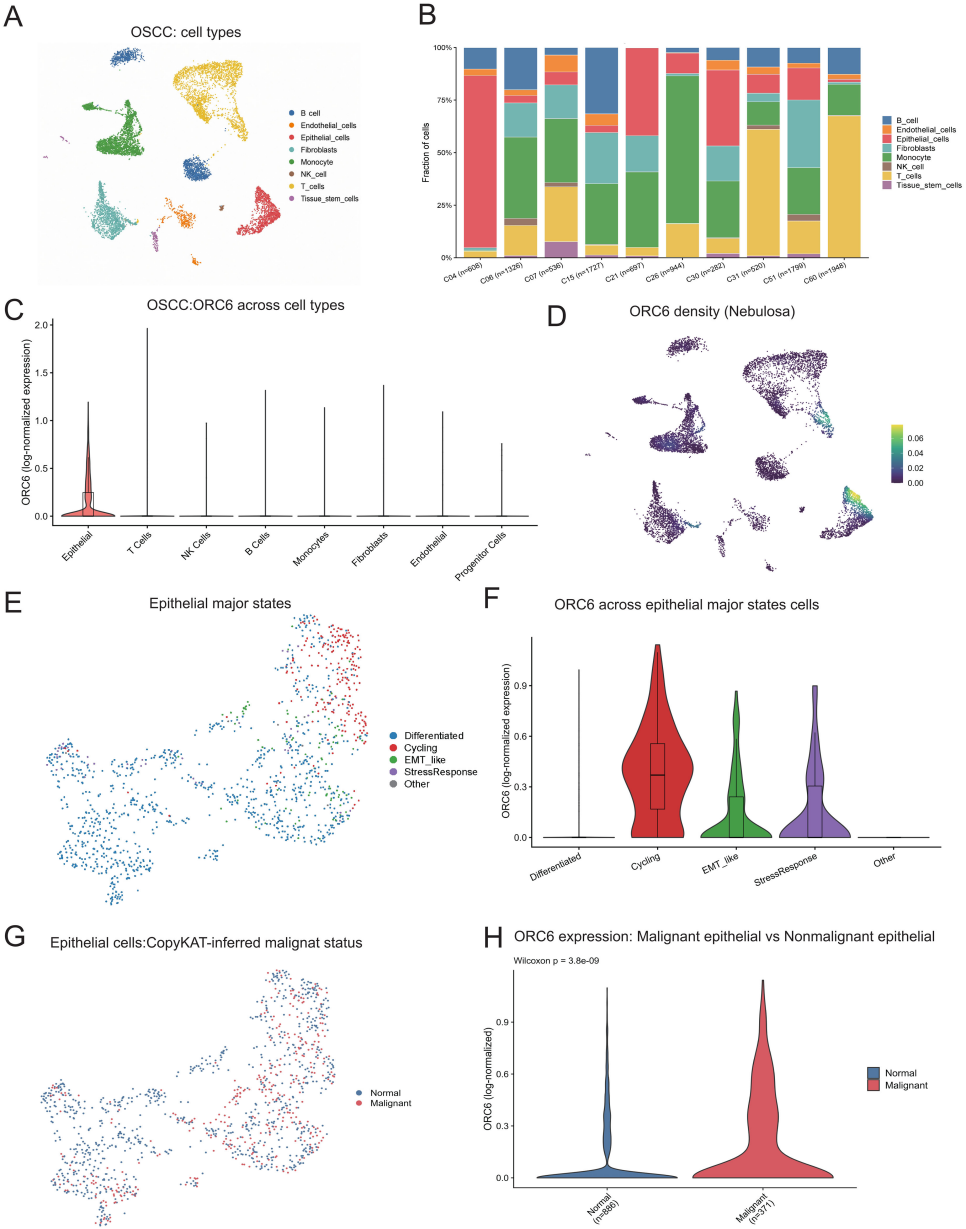
Single-cell mapping localizes ORC6 to malignant epithelium. **(A)** UMAP of major cell types in the OSCC microenvironment. **(B)** Cell-type composition across samples. **(C)** ORC6 expression across major cell types. **(D)** Nebulosa density map of ORC6 expression. **(E)** UMAP of major epithelial program states, including differentiated, cycling, EMT-like, and stress-related states. **(F)** ORC6 expression across epithelial program states. **(G)** CopyKAT-inferred malignant and nonmalignant epithelial cells. **(H)** Comparison of ORC6 expression between malignant and nonmalignant epithelial cells.

To define the cellular context of ORC6 in OSCC, we integrated and annotated the single-cell dataset from GSE181919. UMAP visualization identified the major cell populations in the tumor microenvironment, including epithelial cells, fibroblasts, monocyte/macrophage populations, T cells, B cells, and endothelial cells (**Figure 4A**). Sample-wise composition showed inter-sample variability but preserved the major cellular components expected in OSCC (**Figure 4B**).

ORC6 expression was concentrated mainly in the epithelial compartment and remained low in most immune and stromal populations (**Figure 4C**). Nebulosa density mapping further localized the strongest ORC6 signal to epithelial clusters (**Figure 4D**). Within the epithelial lineage, cells could be broadly grouped into differentiated, cycling, EMT-like, and stress-response states (**Figure 4E**), and ORC6 expression was highest in the cycling state, while remaining relatively elevated in EMT-like and stress-response states (**Figure 4F**).

Using CopyKAT-based CNV inference, epithelial cells were further classified as diploid or aneuploid (**Figure 4G**). Under this framework, ORC6 expression was significantly higher in CopyKAT-defined malignant epithelial cells than in CopyKAT-diploid epithelial cells (**Figure 4H**). Together, these data suggest that the bulk-level increase in ORC6 primarily reflects a malignant epithelial cell-intrinsic signal. Additional supporting analyses are shown in **Supplementary Figure 5**.

### 3.4 The ORC6-high malignant epithelial state is characterized by replication and cell-cycle activation, with overall preservation after cell-cycle regression

**Figure 5:**
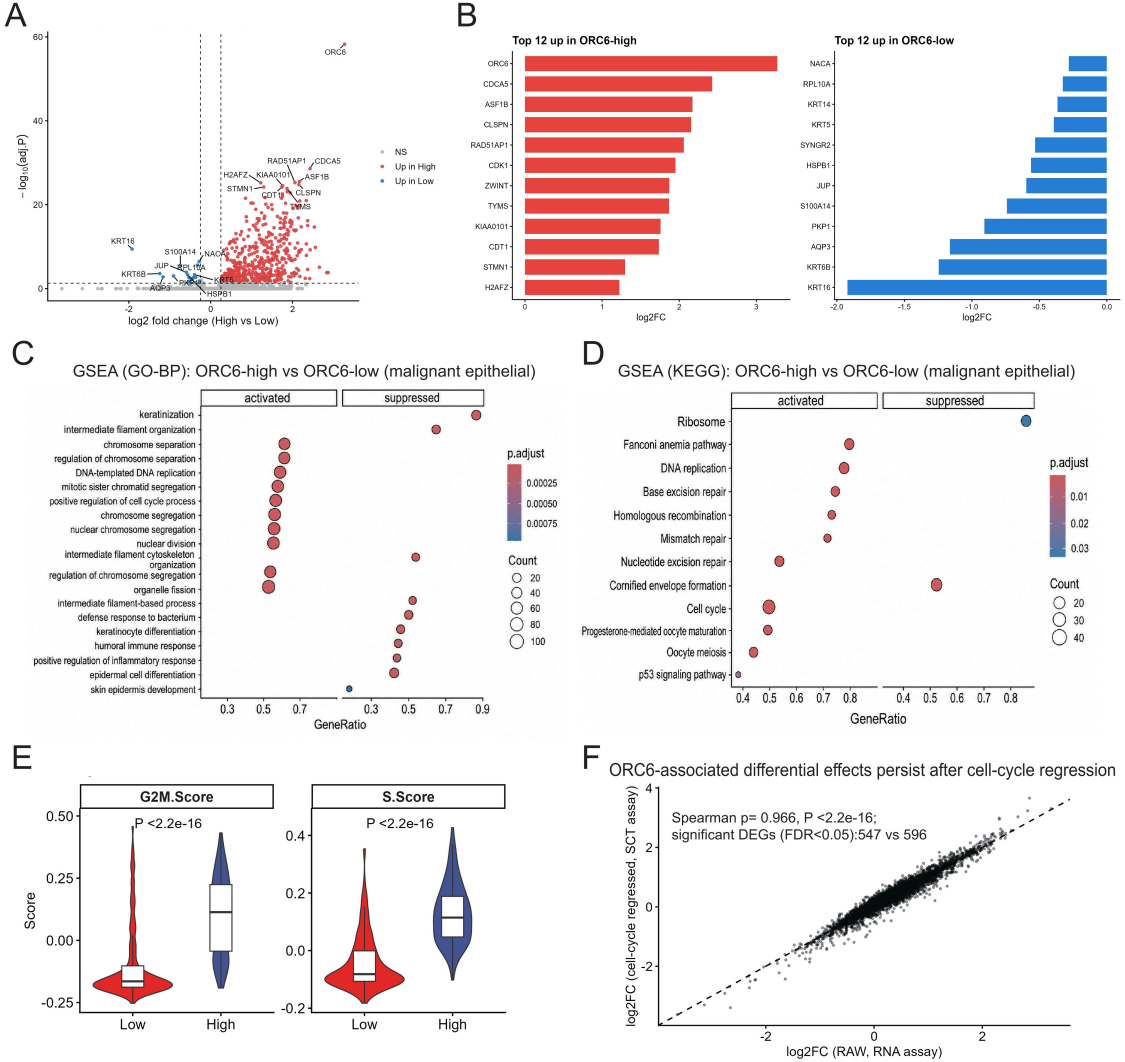
ORC6 stratification identifies a replication-active malignant epithelial state that remains stable after cell-cycle correction. **(A)** Volcano plot of differential expression between ORC6-high and ORC6-low malignant epithelial cells. **(B)** Representative differentially expressed genes. **(C)** Key enriched pathways summarized from the main analysis; dot size indicates -log10(FDR) and color indicates normalized enrichment score (NES). **(D)** Donor-paired pseudobulk Hallmark GSEA validation. **(E)** Comparison of S and G2M scores between ORC6-high and ORC6-low malignant epithelial cells. **(F)** Concordance of differential effects before and after S/G2M regression. Regression efficacy is shown in **Supplementary Figure 6**.

Within CopyKAT-defined malignant epithelial cells, ORC6-high and ORC6-low subgroups were compared by differential expression analysis. Genes upregulated in the ORC6-high group were dominated by DNA replication- and cell-cycle-related factors, whereas genes relatively enriched in the ORC6-low group were more related to differentiation and immune-associated features (**Figures 5A,B**). This pattern supports the notion that ORC6-high malignant epithelial cells occupy a replication-active transcriptional state.

Pathway analysis further reinforced this interpretation. In malignant epithelial cells, ORC6-high was associated with enrichment of replication- and proliferation-related programs, including HALLMARK_E2F_TARGETS and HALLMARK_G2M_CHECKPOINT, whereas differentiation-related processes were reduced and several immune-related pathways, including interferon response and antigen-processing/presentation programs, showed negative shifts (**Figure 5C**).

To reduce pseudoreplication and assess whether these differences were preserved at the donor level, we performed donor-paired pseudobulk validation. Hallmark GSEA showed persistent enrichment of proliferative programs such as DNA_REPAIR, E2F_TARGETS, G2M_CHECKPOINT, and MITOTIC_SPINDLE in ORC6-high samples, whereas INTERFERON_GAMMA_RESPONSE and INTERFERON_ALPHA_RESPONSE were negatively enriched (**Figure 5D**). Thus, the ORC6-high state was not limited to cell-level fluctuations but could still be detected after sample-level aggregation.

Because ORC6 is biologically linked to DNA replication and cell-cycle progression, we then treated cell cycle as a potential confounder. As expected, ORC6-high cells had higher G2M and S scores (**Figure 5E**). After regression of S and G2M scores within the SCTransform framework, the overall differential pattern remained largely preserved. Effect sizes before and after regression were highly concordant (**Figure 5F**), indicating that ORC6-associated transcriptional differences were not abolished by cell-cycle adjustment. Additional analyses quantifying the reduction of gene-cell-cycle covariance after regression are shown in **Supplementary Figure 6**.

### 3.5 Virtual knockdown and cross-level validation support an ORC6-associated replication program

**Figure 6:**
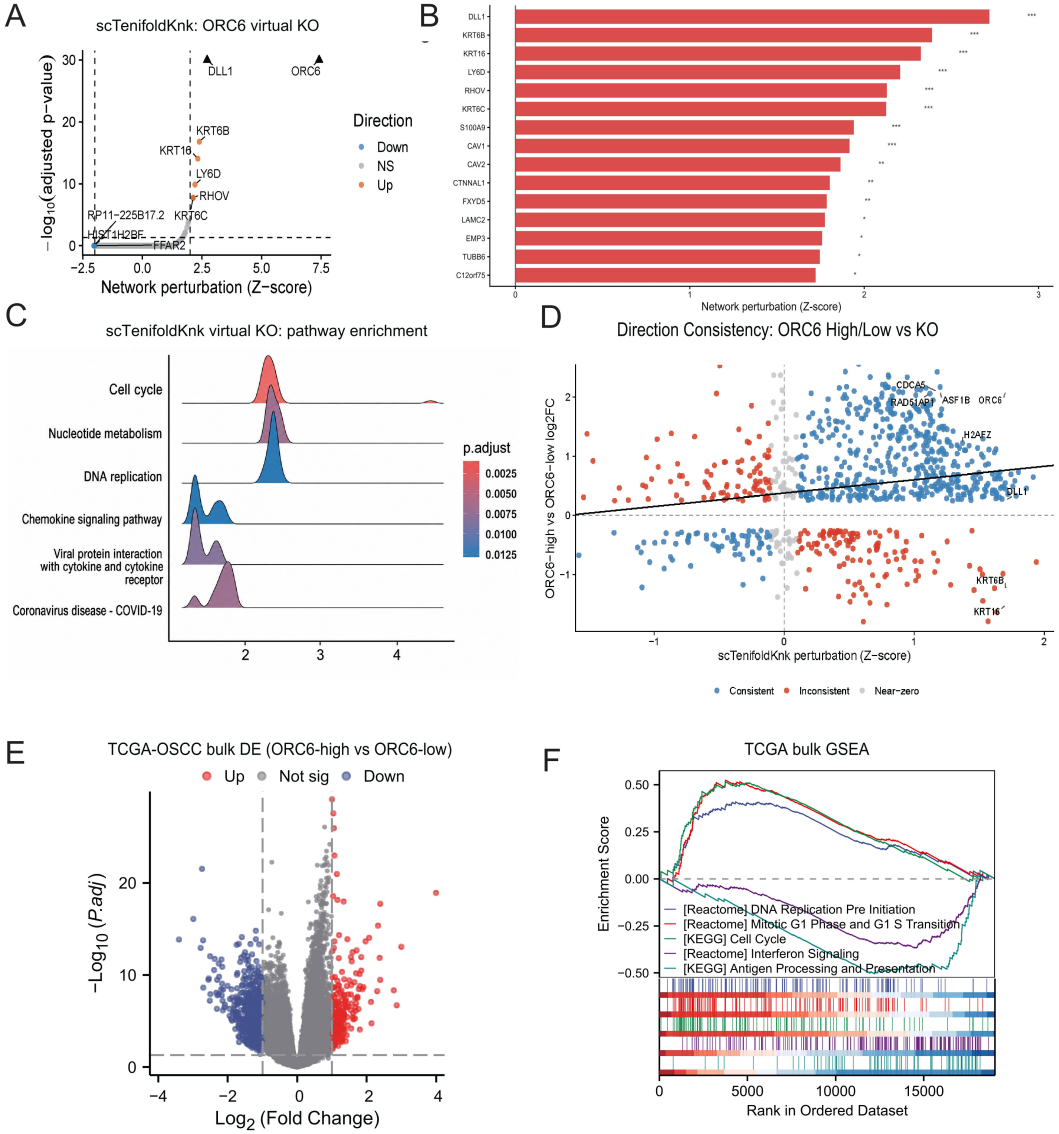
Virtual ORC6 perturbation and TCGA bulk analysis support the same replication-centered program. **(A)** Perturbation strength and significance after virtual ORC6 knockout by scTenifoldKnk. **(B)** Representative perturbed genes. **(C)** Pathway enrichment overview of the perturbation signature. **(D)** Directional concordance between observed ORC6-high/ORC6-low effects and virtual perturbation effects. **(E)** Bulk differential expression between ORC6-high and ORC6-low tumors in TCGA-OSCC. **(F)** GSEA of key pathways in TCGA-OSCC bulk validation.

To further assess whether the ORC6-high malignant epithelial state was linked to a trackable functional dependency, we performed in silico ORC6 knockdown using scTenifoldKnk in malignant epithelial cells. Virtual perturbation induced marked network-level changes (**Figure 6A**), and the most affected genes were enriched for DNA replication and cell-cycle regulators (**Figure 6B**). Pathway analysis of the perturbation signature again highlighted DNA-replication and cell-cycle processes as the major affected programs (**Figure 6C**).

We then compared the perturbation effect with the ORC6-high versus ORC6-low differential signature. The two showed overall directional concordance at the gene level (**Figure 6D**), indicating that the transcriptional state associated with high ORC6 expression aligned with the perturbation pattern obtained by virtual ORC6 depletion.

To examine whether this replication-associated pattern could also be detected at the patient level, we performed ORC6-high versus ORC6-low differential analysis in TCGA-OSCC bulk samples. Bulk-level differential expression and GSEA again pointed to enhanced replication- and cell-cycle-related programs, together with directional changes in several immune and signaling pathways (**Figures 6E,F**). These findings provide cross-level support for an ORC6-associated replication-active transcriptional program.

### 3.6 Bulk immune analyses reveal proliferation-adjusted inverse associations between ORC6 and selected immune-related features in TCGA-OSCC

**Figure 7:**
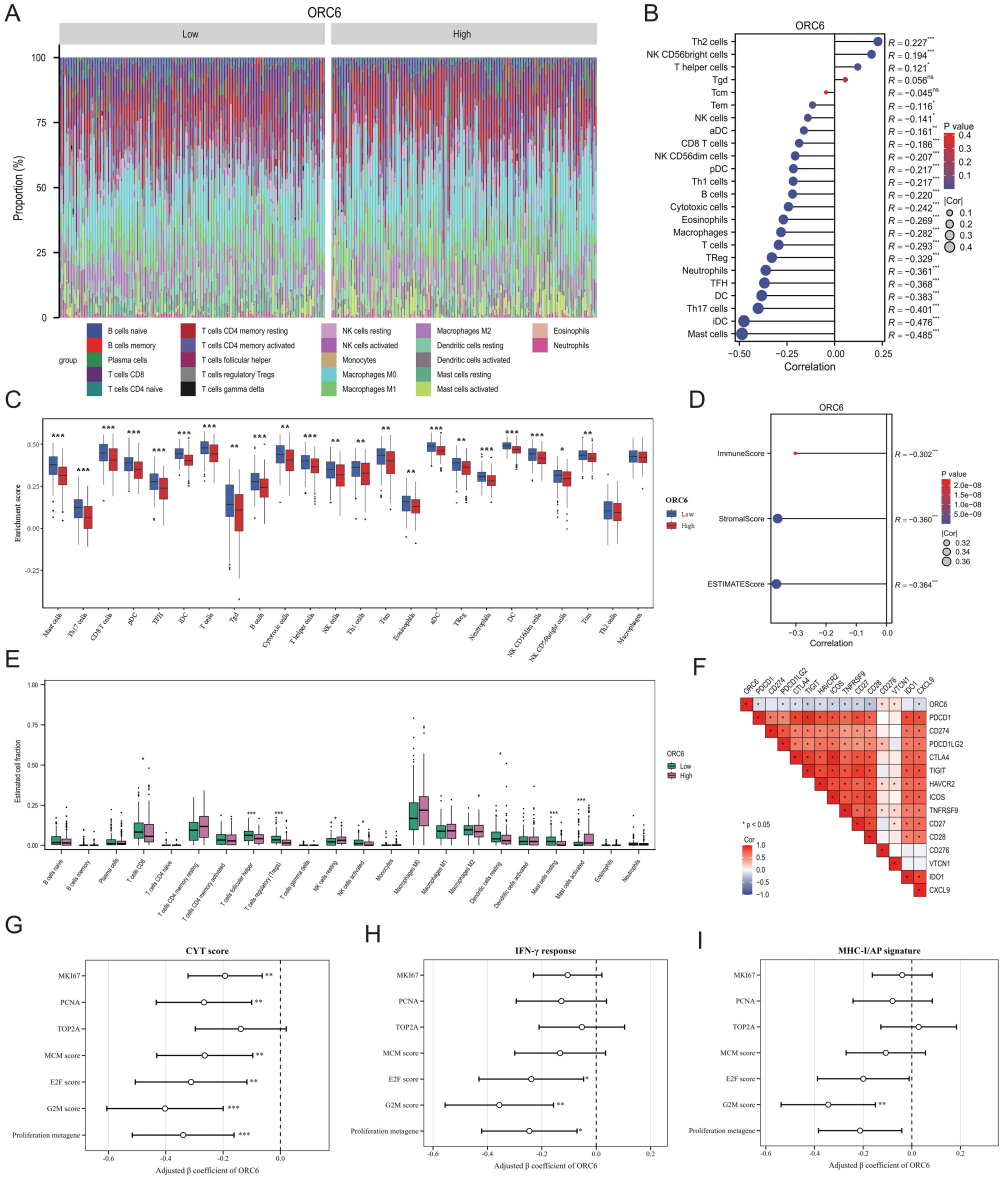
Inverse association between ORC6 expression and immune-related features in bulk OSCC. **(A)** CIBERSORT-based overview of the relative proportions of 22 immune cell types in ORC6-low and ORC6-high tumors from the strictly defined TCGA-OSCC cohort. Each bar represents one tumor sample. **(B)** Spearman correlation analysis between ORC6 expression and ssGSEA-derived immune cell/state scores. Dot color indicates the P value, and dot size indicates the absolute correlation coefficient. **(C)** Grouped comparison of ssGSEA-derived immune enrichment scores between ORC6-low and ORC6-high tumors. **(D)** Spearman correlation analysis between ORC6 expression and ESTIMATE-derived ImmuneScore, StromalScore, and ESTIMATEScore. Dot color indicates the P value, and dot size indicates the absolute correlation coefficient. **(E)** Grouped comparison of CIBERSORT-estimated immune cell fractions between ORC6-low and ORC6-high tumors. **(F)** Correlation heatmap showing the association between ORC6 and selected immune checkpoint or immunoregulatory genes. **(G–I)** Proliferation-adjusted associations between ORC6 expression and three representative immune-related features, including CYT score **(G)**, IFN-γ response **(H)**, and MHC-I/antigen-presentation signature **(I)**. Adjusted β coefficients of ORC6 were extracted from multivariable linear models adjusted separately for MKI67, PCNA, TOP2A, MCM score, E2F score, G2M score, or the proliferation metagene. Asterisks indicate statistical significance. *p<0.05.

Having defined an ORC6-associated malignant epithelial state at single-cell resolution, we next asked whether tumors with higher ORC6 expression were associated with a less inflamed tissue-level transcriptional context in the strictly defined TCGA-OSCC cohort. Complementary deconvolution and scoring approaches were used, including CIBERSORT, ssGSEA/GSVA, and ESTIMATE. CIBERSORT-based analysis showed heterogeneous changes across immune cell subsets, while ssGSEA-based analyses indicated that ORC6 was negatively correlated with multiple immune-related cell states and immune-context features (**Figures 7A–C**). ESTIMATE analysis further showed inverse correlations between ORC6 and immune-related microenvironmental scores, including ImmuneScore, StromalScore, and ESTIMATEScore (**Figure 7D**). ORC6 expression was also correlated with several immune checkpoint and immunoregulatory genes, supporting a broad association between ORC6 expression and immune-related transcriptional context (**Figure 7F**).

Because ORC6 is intrinsically linked to DNA replication and cell-cycle progression, we next directly compared ORC6 with canonical proliferation markers and proliferation-related signatures. ORC6 was strongly and positively correlated with MKI67, PCNA, TOP2A, MCM score, E2F score, G2M score, and the proliferation metagene (**Supplementary Figure 3A**). Representative scatter plots further showed positive correlations between ORC6 and MKI67, TOP2A, PCNA, MCM score, and E2F score (**Supplementary Figures 3B–F**). These findings confirmed that ORC6 is closely embedded in a proliferation-related transcriptional program and justified the need for proliferation-adjusted analyses.

We therefore fitted multivariable models incorporating multiple proliferation-related covariates. Across models adjusted for MKI67, PCNA, TOP2A, MCM score, E2F score, G2M score, or the proliferation metagene, ORC6 retained directionally negative associations with CYT score, IFN-γ response, and MHC-I/antigen-presentation signature, although the magnitude and statistical significance varied depending on the proliferation covariate used (**Figures 7G–I**). To further reduce the influence of extreme values, we repeated selected analyses after winsorization. Winsorized residual analyses showed that ORC6 remained negatively associated with proliferation-adjusted CYT score and IFN-γ response, whereas the association with MHC-I/antigen-presentation score showed a weaker but directionally consistent trend (**Supplementary Figures 4A–C**). These findings support a correlative inverse association between ORC6 expression and selected immune-related transcriptional features in bulk OSCC data after accounting for proliferative activity.

To further evaluate whether the prognostic association of ORC6 could be distinguished from canonical proliferation-related markers, we performed proliferation-adjusted Cox sensitivity analyses. In the OS-valid TCGA-OSCC cohort, ORC6-high patients showed poorer OS than ORC6-low patients using median-based stratification (**Supplementary Figure 4D**). In Cox models adjusted for pathologic T stage, pathologic N stage, age, lymph node neck dissection, and one proliferation-related covariate at a time, the adverse association of ORC6 with OS remained directionally consistent across all models (**Supplementary Figure 4E**). The association retained nominal significance after adjustment for MKI67, PCNA, and MCM score, whereas adjustment for TOP2A, E2F score, G2M score, or the broader proliferation metagene attenuated statistical significance. These results indicate that ORC6 is strongly proliferation-linked and should not be interpreted as fully independent of proliferation. Rather, ORC6 marks a replication-active epithelial state whose immune and prognostic associations are partially, but not completely, separable from broader proliferation programs.

### 3.7 Transcript-based CellChat inference suggests hypothesis-generating epithelial–T-cell interaction patterns associated with the ORC6-high state

**Figure 8:**
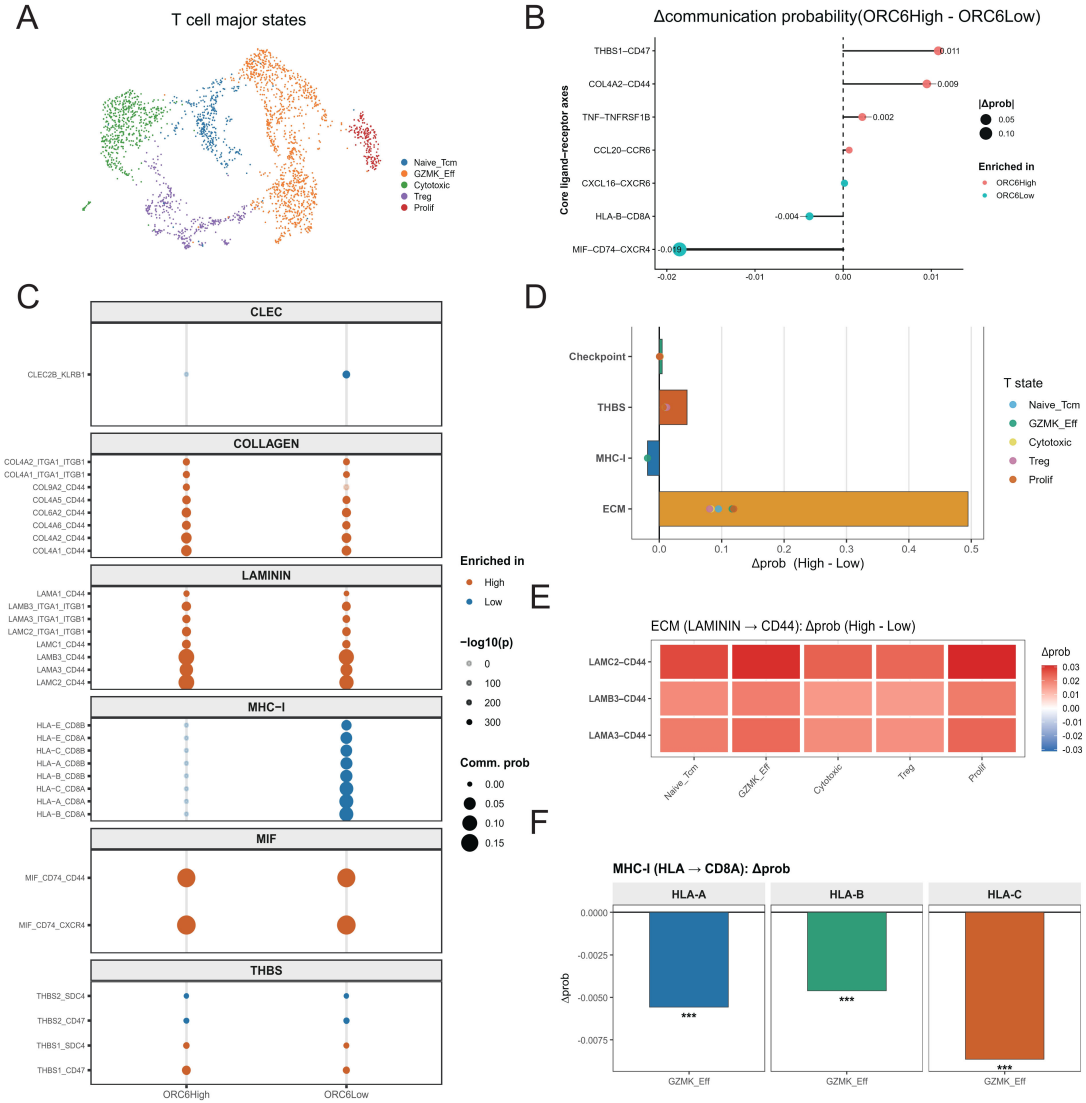
Transcript-based CellChat inference suggests hypothesis-generating epithelial–T-cell interaction patterns associated with the ORC6-high malignant epithelial state. **(A)** Overview of major T-cell states. **(B)** Representative ligand-receptor pairs showing differential communication strength between ORC6-high and ORC6-low conditions. **(C)** Pathway-level summary of differential interaction pairs across T-cell states. **(D)** Axis-level summary of the direction and magnitude of communication changes. **(E)** Δprob heatmap for a representative ECM-CD44 interaction axis across T-cell states. **(F)** Representative MHC-I-related interaction view (HLA-CD8). Δprob = prob(ORC6High) - prob(ORC6Low); Δprob > 0 indicates relative enrichment in ORC6-high, whereas Δprob< 0 indicates relative enrichment in ORC6-low. ***P < 0.001.

Given that the bulk analyses indicated weaker T-cell-related effector features in ORC6-high tumors, including reduced cytolytic activity and IFN-γ-associated signatures, we used CellChat as a hypothesis-generating framework to examine epithelial–T-cell communication at single-cell resolution. We first defined the major T-cell states in the OSCC single-cell dataset (**Figure 8A**; **Supplementary Figure 7A**) and then compared inferred communication patterns between ORC6-high versus ORC6-low malignant epithelial cells and these T-cell subsets. At the ligand-receptor level, representative differential pairs suggested that ORC6-high epithelial cells preferentially engaged ECM- and adhesion-related interaction modules, particularly within COLLAGEN- and LAMININ-related pathways, whereas transcript-inferred MHC-I-related interaction axes showed relative attenuation under the ORC6-high condition (**Figure 8B**). Pathway-level summaries supported the same directional pattern and also indicated concurrent changes in additional immune-regulatory pathways, including THBS, MIF, and CLEC-related modules (**Figure 8C**).

At the axis level, Δprob summaries further showed that ECM/adhesion-related and selected immune-regulatory interaction axes tended to shift upward, whereas MHC-I-related axes tended to shift downward in ORC6-high epithelial cells (**Figure 8D**). Representative examples illustrated this pattern more directly: ECM-CD44-related interactions showed directionally increased signaling across T-cell states (**Figure 8E**), whereas the HLA-CD8 axis, used here as an example of MHC-I-mediated recognition, showed reduced interaction strength (**Figure 8F**).

Expression-level support for key ligands and receptors is provided in **Supplementary Figures 7D, E**, and additional state-stratified interaction patterns are shown in **Supplementary Figure 7**. Overall, these analyses suggest a directional shift toward ECM/adhesion-related interaction patterns together with weaker MHC-I-related interaction axes in the ORC6-high condition. Because these inferences are transcript-based, they should be interpreted as contextual support rather than direct evidence of altered intercellular signaling.

### 3.8 ORC6 knockdown suppresses cell growth, invasion, and migration in CAL27 cells

**Figure 9:**
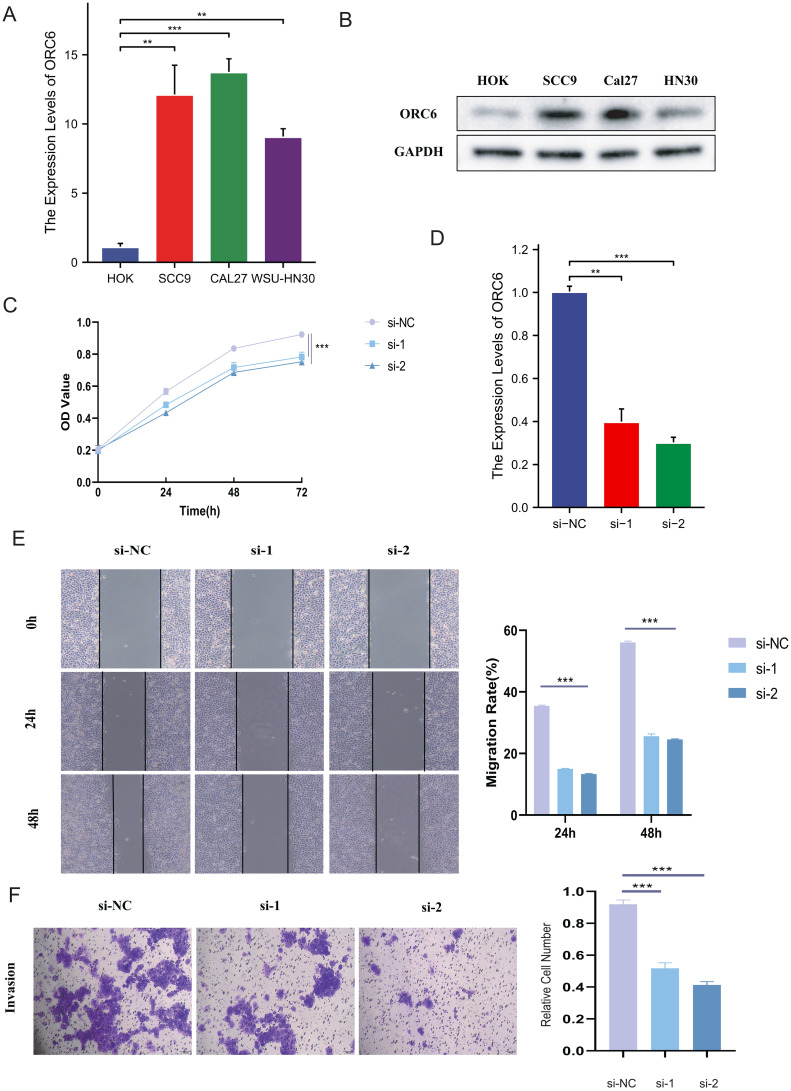
ORC6 knockdown suppresses cell growth, migration, and invasion in CAL27 cells. **(A)** qRT-PCR analysis of ORC6 expression in HOK and OSCC cell lines. **(B)** Representative Western blot showing ORC6 protein expression. **(C)** CCK-8 assay after ORC6 knockdown. **(D)** qRT-PCR validation of ORC6 knockdown efficiency in CAL27 cells. **(E)** Representative wound-healing images and quantification of migration rate. **(F)** Representative Transwell invasion images and quantification of relative invaded cell numbers.Data are shown as mean ± SD from three independent biological replicates unless otherwise indicated. **P < 0.01, ***P < 0.001.

To further verify the upregulation of ORC6 indicated by the transcriptomic analyses, we examined ORC6 expression at the cellular level in normal human oral keratinocytes (HOK) and OSCC cell lines, including SCC9, CAL27, and WSU-HN30. qRT-PCR showed that ORC6 mRNA levels were significantly higher in OSCC cell lines than in HOK cells. Western blotting further confirmed elevated ORC6 protein expression in OSCC cell lines, with relatively higher expression observed in SCC9 and CAL27 cells (**Figures 9A,B**).

To investigate the functional role of ORC6, CAL27 cells were transfected with two independent ORC6-targeting siRNAs, siORC6–1 and siORC6-2, or the negative-control siRNA, siNC. Transcript-level knockdown efficiency was confirmed by qRT-PCR. Functionally, CCK-8 assays showed that ORC6 knockdown reduced CAL27 cell growth over time, with both siORC6–1 and siORC6–2 resulting in lower OD450 values than the siNC group (**Figure 9C**). Transwell assays further showed that ORC6 knockdown decreased the invasive capacity of CAL27 cells, as reflected by fewer invading cells in both siORC6 groups than in the siNC group (**Figure 9F**). Similarly, wound-healing assays demonstrated that ORC6 silencing impaired migratory ability, with lower wound-closure rates observed at 24 h and 48 h after knockdown (**Figure 9E**). Together, these results support a functional role for ORC6 in maintaining cell growth, invasion, and migration in CAL27 cells.

### 3.9 ORC6 knockdown increases antigen-presentation-associated features and reduces S-phase fraction in CAL27 cells

**Figure 10:**
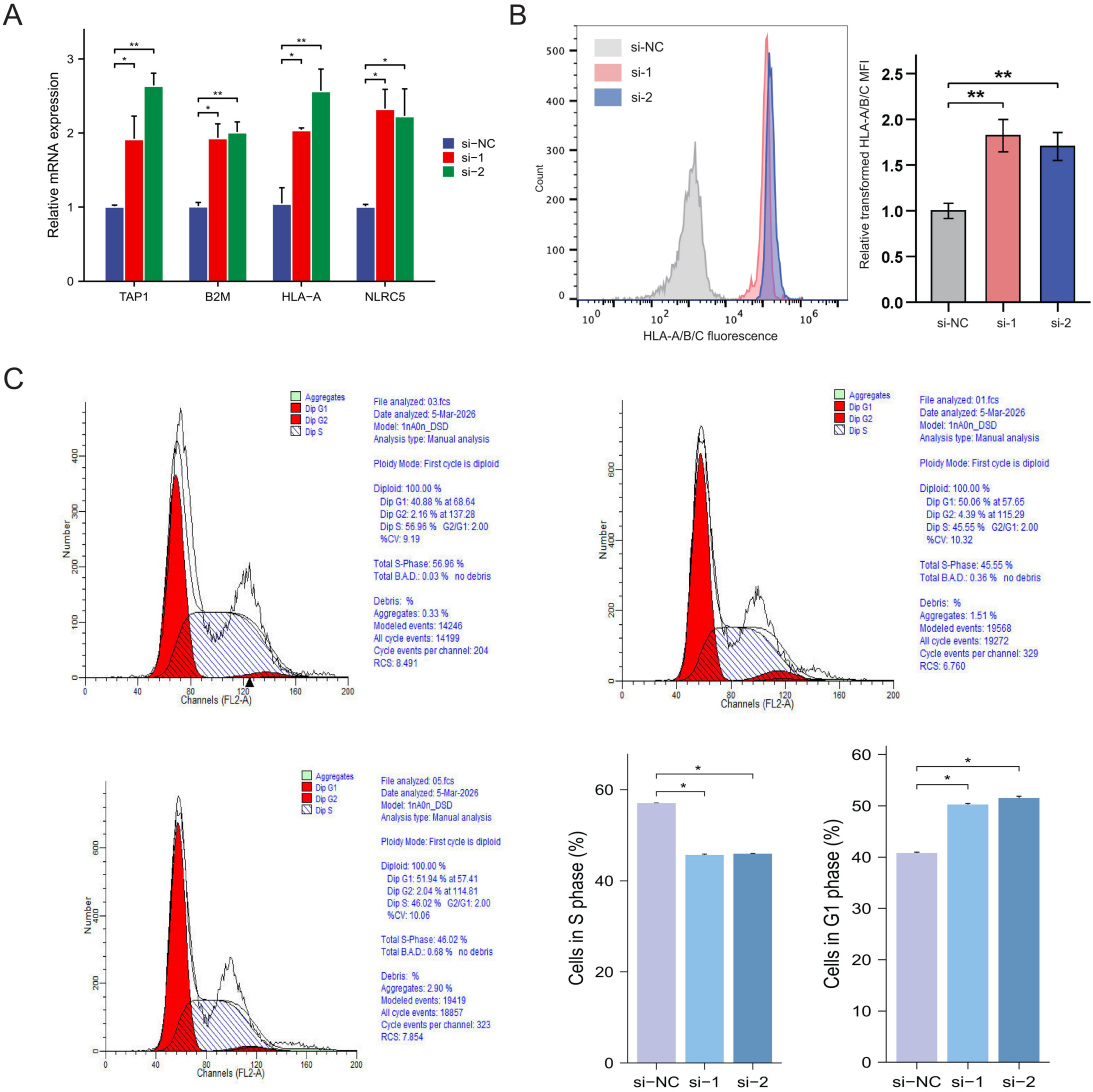
ORC6 knockdown reduces S-phase fraction and increases antigen-presentation-associated features in CAL27 cells. **(A)** qRT-PCR analysis of antigen-presentation-associated genes, including TAP1, B2M, HLA-A, and NLRC5, in CAL27 cells transfected with siNC, si-1, or si-2. **(B)** Representative flow cytometry histograms showing surface HLA-A/B/C expression and quantification of relative transformed HLA-A/B/C mean fluorescence intensity (MFI) in CAL27 cells after ORC6 knockdown. MFI values were normalized to the siNC group. **(C)** Representative flow cytometric cell-cycle profiles and quantification of the percentages of cells in G1 and S phases in CAL27 cells transfected with siNC, si-1, or si-2.Data are shown as mean ± SD from three independent biological replicates unless otherwise indicated. *P < 0.05, **P < 0.01.

Given the inverse association between ORC6 expression and immune-related transcriptional features observed in the integrative analyses, we next examined whether ORC6 knockdown was accompanied by changes in tumor cell-intrinsic antigen-presentation-associated genes. qRT-PCR analysis showed that TAP1, B2M, HLA-A, and NLRC5 were increased to varying degrees in ORC6-silenced CAL27 cells compared with the siNC group, with most comparisons reaching nominal statistical significance (**Figure 10A**). These findings suggest that ORC6 knockdown is accompanied by increased antigen-presentation-associated transcriptional features in CAL27 cells.

Because transcript-level changes alone do not establish protein-level alterations in MHC-I-related surface expression, we further assessed surface HLA-A/B/C expression by flow cytometry. Representative flow cytometry histograms showed a rightward shift of HLA-A/B/C fluorescence in both siORC6–1 and siORC6–2 groups compared with the siNC group (**Figure 10B**). Quantification of relative transformed HLA-A/B/C mean fluorescence intensity (MFI), normalized to the siNC group, showed that surface HLA-A/B/C expression was significantly increased after ORC6 knockdown in both siORC6–1 and siORC6–2 cells (**Figure 10B**). These results provide protein-level support that ORC6 knockdown is accompanied by increased surface MHC-I-related expression in CAL27 cells.

In parallel, cell-cycle analysis indicated that ORC6 knockdown altered cell-cycle distribution in CAL27 cells. Compared with the siNC group, both siORC6–1 and siORC6–2 increased the proportion of cells in G1 phase and reduced the proportion of cells in S phase (**Figure 10C**). Specifically, the siNC group showed approximately 40.9% of cells in G1 phase and 57.0% in S phase, whereas the siORC6–1 and siORC6–2 groups showed increased G1 phase fractions of approximately 50.0% and 51.9%, respectively, together with reduced S phase fractions of approximately 45.6% and 46.0%. This pattern is consistent with impaired cell-cycle progression after ORC6 depletion.

Taken together, these *in vitro* results indicate that ORC6 knockdown suppresses malignant cellular phenotypes, reduces S-phase fraction, and is accompanied by increased antigen-presentation-associated transcripts and surface HLA-A/B/C expression in CAL27 cells. These findings provide tumor cell-intrinsic transcript- and protein-level support for the immune-related associations identified in the integrative analyses, although they should be interpreted as increased surface MHC-I-related expression rather than direct evidence of restored functional antigen presentation or enhanced immune-cell-mediated recognition.

## 4 Discussion

In this study, we focused on the malignant epithelial compartment of OSCC and identified ORC6 as a marker of a replication-active tumor-cell state that was reproducibly associated with immune-low features across bulk, single-cell, and experimental analyses. Several aspects of this pattern are notable. First, ORC6 expression was preferentially enriched in malignant epithelial cells rather than broadly distributed across the microenvironment. Second, the ORC6-high state was consistently linked to DNA replication and cell-cycle programs. Third, this state also showed weaker immune-related features, with particularly consistent attenuation of MHC-I/antigen-presentation signals. Taken together, these findings support a model in which ORC6 is strongly proliferation-linked but may serve as a marker of a replication-active malignant epithelial program associated with immune-low transcriptional features (20).

From a study-design perspective, this work addresses two recurring interpretive problems in OSCC and broader HNSCC research. One is the frequent reliance on anatomically mixed HNSCC cohorts, which can obscure disease-specific links between tumor-cell programs and the local immune context (21). The other is the tendency to treat replication- and cell-cycle-related genes as interchangeable proliferation surrogates, thereby limiting their biological interpretability in studies of tumor-immune coupling (22). Our cross-level design was intended to address both issues by anchoring the analysis in OSCC, resolving cell states at single-cell resolution, and then testing whether the observed associations remained directionally stable after accounting for cell-cycle and proliferation-related confounding.

Biologically, one of the central observations was that ORC6-high malignant epithelial cells combined strong replication and cell-cycle activity with weaker immune-related transcriptional features. This finding is consistent with the established role of ORC6 in replication licensing and origin assembly (10), as well as with prior literature linking ORC6 upregulation to proliferative advantage in multiple malignancies (12). At the same time, the coexistence of a replication-active program with reduced IFN-related and antigen-presentation-related signals is conceptually important. We do not interpret this pattern as evidence against the broader framework in which replication stress, genomic instability, or aberrant nucleic-acid sensing may activate innate immune pathways (23–25). Rather, our data suggest that in the ORC6-high OSCC epithelial state, replication-linked activity is not accompanied by a correspondingly strengthened immune-alert program at the transcriptomic level. One plausible interpretation is that, within the ORC6-high OSCC epithelial state, replication-associated activity may coexist with relatively attenuated interferon- and antigen-presentation-related transcriptional features. At present, however, our data support this as a state-level association and hypothesis-generating framework rather than evidence of a defined causal immune-regulatory mechanism.

Among the immune-related findings, the relative consistency of reduced MHC-I/antigen-presentation signatures may be especially informative. Compared with broader inflammatory readouts, antigen-processing and antigen-presentation programs are more directly related to the ability of malignant epithelial cells to remain immunologically visible to cytotoxic lymphocytes (26, 27). In our analyses, this signal persisted directionally across single-cell, pseudobulk, and bulk-level assessments, and selected antigen-presentation-related transcripts also increased after ORC6 knockdown in CAL27 cells. These results do not establish that ORC6 directly suppresses antigen presentation or functionally alters tumor-cell recognition by immune effectors. They do, however, support the possibility that the ORC6-associated epithelial state is linked to attenuated antigen-presentation-associated transcriptional features within malignant epithelial cells. This distinction is important because it places the most consistent immune-related observation in antigen-presentation-associated programs rather than in generalized immune depletion alone.

The bulk immune analyses were broadly aligned with this interpretation. Higher ORC6 expression was associated with lower immune-related scores, weaker effector T-cell features, and reduced IFN-γ response, cytolytic activity, and MHC-I/antigen-presentation signatures. Importantly, these trends remained detectable after accounting for proliferation, which argues against a purely trivial explanation based on highly cycling tumors alone. This does not eliminate residual confounding, nor does it prove that ORC6 itself is the causal determinant of immune deprivation. Nevertheless, the directional persistence of selected associations after adjustment supports the interpretation that ORC6 indexes a biologically coherent replication-active epithelial state associated with immune-low transcriptional features.

Transcript-based CellChat inference provided additional hypothesis-generating context for this interpretation. ORC6-associated malignant epithelial states showed a relative shift toward ECM- and adhesion-related outgoing interaction patterns together with weaker transcript-inferred MHC-I-related interaction axes (28, 29). This configuration is broadly compatible with current models in which extracellular matrix remodeling, stromal organization, and cancer-associated fibroblast-related programs contribute to immune exclusion in OSCC and HNSCC (30–32). It is also consistent with the broader concept that anti-tumor immunity is shaped by a composite cancer-immune set point rather than by a single pathway (33). We emphasize, however, that ligand-receptor inference does not directly establish functional intercellular signaling. Accordingly, these findings are best interpreted as contextual support for the possibility that the ORC6-associated epithelial state may reside within, or contribute to, a microenvironmental configuration that is consistent with a less inflamed tumor context.

To move beyond transcriptomic association alone, we incorporated both in silico perturbation and wet-lab validation. Virtual ORC6 knockout suggested substantial disruption of DNA replication- and cell-cycle-related networks, and the inferred perturbation pattern showed directional opposition to the observed ORC6-high transcriptional program. *In vitro*, ORC6 knockdown in CAL27 cells reduced growth, migration, and invasion, and shifted the cell-cycle distribution toward G0/G1 with a corresponding decrease in S phase. These results support the interpretation that ORC6 is functionally linked to malignant epithelial phenotypes *in vitro*, while the extent to which this reflects broader *in vivo* tumor behavior requires further validation (34). At the same time, the immune-related *in vitro* findings should still be interpreted cautiously. ORC6 knockdown increased selected antigen-presentation-associated transcripts and was accompanied by increased surface HLA-A/B/C expression, providing tumor cell-intrinsic transcript- and protein-level support for the computational association. However, these data do not establish functional antigen presentation, peptide loading efficiency, or enhanced recognition and killing by immune effector cells. Therefore, the immune-related findings remain supportive but not mechanistically definitive.

From a translational perspective, our findings suggest that ORC6 may be useful as a candidate marker of a biologically defined OSCC epithelial state characterized by replication-linked activity and an immune-low transcriptional context. This framing may be more informative than viewing ORC6 solely as a proliferation-associated gene, because it links tumor-cell-intrinsic state biology with features of immune context that could be relevant to stratification. Even so, the present study does not demonstrate that ORC6 is independently actionable as a therapeutic target, nor does it establish that ORC6 expression can directly predict response to immunotherapy. Those questions will require dedicated validation in clinically annotated cohorts and more direct functional studies.

This study has several limitations. The clinical and transcriptomic analyses relied largely on retrospective public datasets, and the prognostic relevance of ORC6 should be further tested in independent external and prospectively curated cohorts. Although multiple strategies were used to address cell-cycle- and proliferation-related confounding, such confounding cannot be fully excluded. In addition, the immune-related conclusions remain strongest at the level of association and transcriptomic inference. The current *in vitro* experiments support a tumor-cell-intrinsic role for ORC6 in malignant phenotypes and provide preliminary support for antigen-presentation-related differences, but they do not establish causal remodeling of the broader immune microenvironment. These limitations define clear directions for future work while preserving the central observation that ORC6 marks a replication-active OSCC malignant epithelial state associated with reproducible immune-low transcriptional features.

## 5 Conclusions

ORC6 marks a replication-active malignant epithelial state in OSCC and is associated with attenuated immune-related features inferred from bulk and single-cell analyses, particularly antigen-presentation-related programs. The available functional data support a role for ORC6 in malignant epithelial fitness and provide preliminary tumor cell-intrinsic support for antigen-presentation-associated transcriptional changes. These findings support ORC6 as a candidate biomarker for biological stratification in OSCC and motivate further mechanistic investigation of how replication-linked tumor-cell states relate to immune context in this disease.

## Data Availability

The datasets presented in this study can be found in online repositories. The names of the repository/repositories and accession number(s) can be found in the article/supplementary material.

## Glossary

ADMET: Absorption, distribution, metabolism, excretion, and toxicity
AUC: Area under the curve
B2M: Beta-2-microglobulin
CCK-8: Cell Counting Kit-8
CI: Confidence interval
CNV: Copy number variation
DSS: Disease-specific survival
ECM: Extracellular matrix
EMT: Epithelial–mesenchymal transition
ESTIMATE: Estimation of STromal and Immune cells in MAlignant Tumors using Expression data
FDR: False discovery rate
GEO: Gene Expression Omnibus
GSEA: Gene set enrichment analysis
GSVA: Gene set variation analysis
HBA: Hydrogen bond acceptor
HBD: Hydrogen bond donor
HLA: Human leukocyte antigen
HNSCC: Head and neck squamous cell carcinoma
HOK: Human oral keratinocytes
HPA: Human Protein Atlas
HR: Hazard ratio
IFN: Interferon
KEGG: Kyoto Encyclopedia of Genes and Genomes
LINCS: Library of Integrated Network-Based Cellular Signatures
MHC-I: Major histocompatibility complex class I
MW: Molecular weight
NES: Normalized enrichment score
NLRC5: NLR family CARD domain containing 5
ORC: Origin recognition complex
ORC6: Origin recognition complex subunit 6
OS: Overall survival
OSCC: Oral squamous cell carcinoma
PCA: Principal component analysis
PDB: Protein Data Bank
PD-1: Programmed cell death protein 1
PFI: Progression-free interval
PI: Propidium iodide
qRT-PCR: Quantitative real-time polymerase chain reaction
ROC: Receiver operating characteristic
SD: Standard deviation
siNC: Negative control small interfering RNA
siORC6: Small interfering RNA targeting ORC6
siRNA: Small interfering RNA
ssGSEA: Single-sample gene set enrichment analysis
SVM-RFE: Support vector machine-recursive feature elimination
TAP1: Transporter associated with antigen processing 1
TCGA: The Cancer Genome Atlas
TME: Tumor microenvironment
TPSA: Topological polar surface area
UMAP: Uniform Manifold Approximation and Projection
UMI: Unique molecular identifier.
