# Supplementary material for: ORC6 marks a replication-active malignant epithelial state and is associated with immune-low features in oral squamous cell carcinoma
Source: Front Oncol. 2026 Jul 21;16:1882429. doi: 10.3389/fonc.2026.1882429 (PMC13433198; doi:10.3389/fonc.2026.1882429)

A

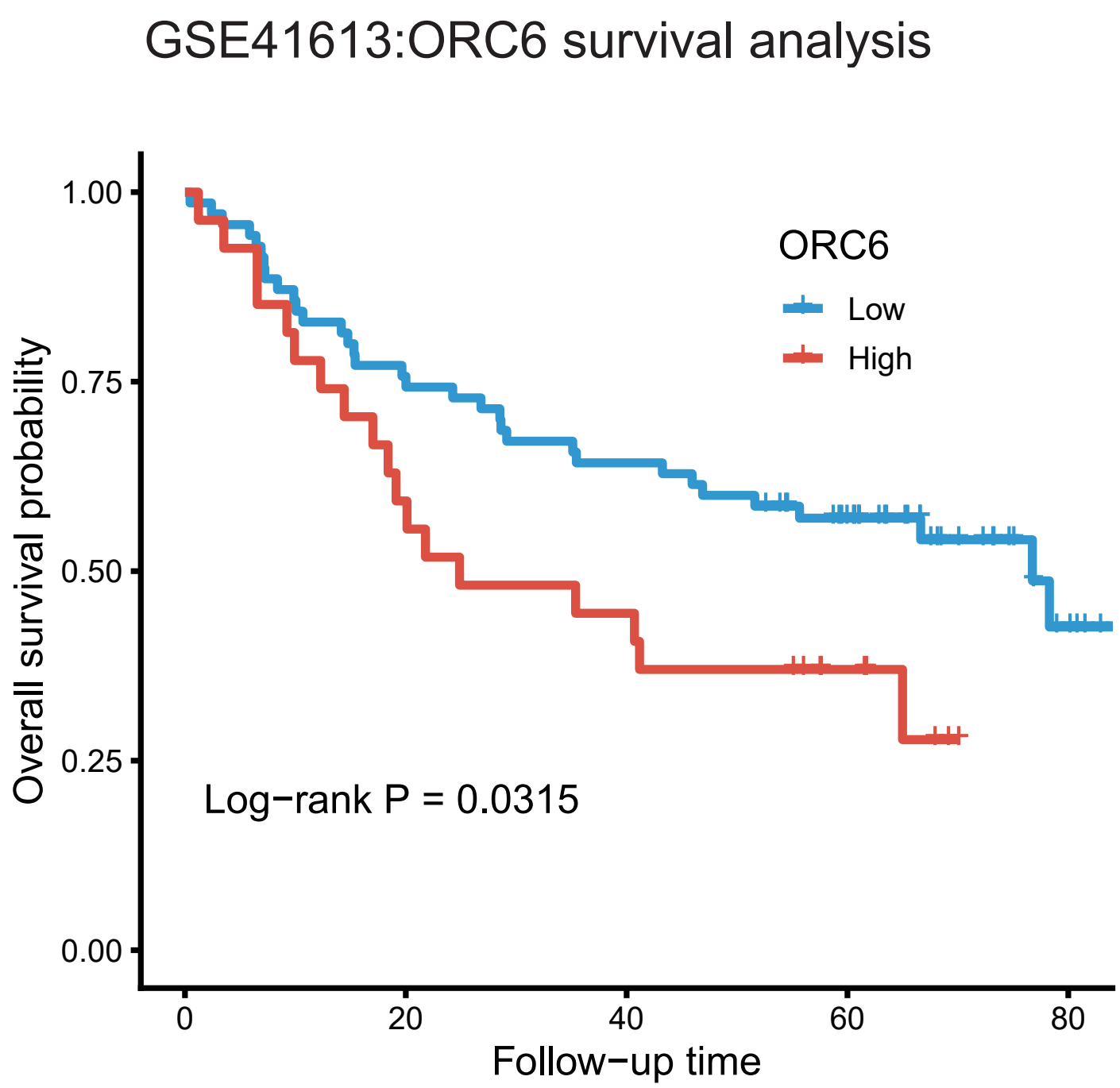

B

Univariable Cox analysis in GSE41613

Variable	N/Events	HR (95% CI)	P value
Tumor stage<br>Stage III–IV	97/51	3.829 (1.959–7.485)	<0.001
ORC6 expression<br>High	97/51	1.879 (1.048–3.369)	0.034
Sex<br>Male	97/51	1.122 (0.621–2.029)	0.702
Age<br>>50 years	97/51	0.739 (0.423–1.293)	0.289

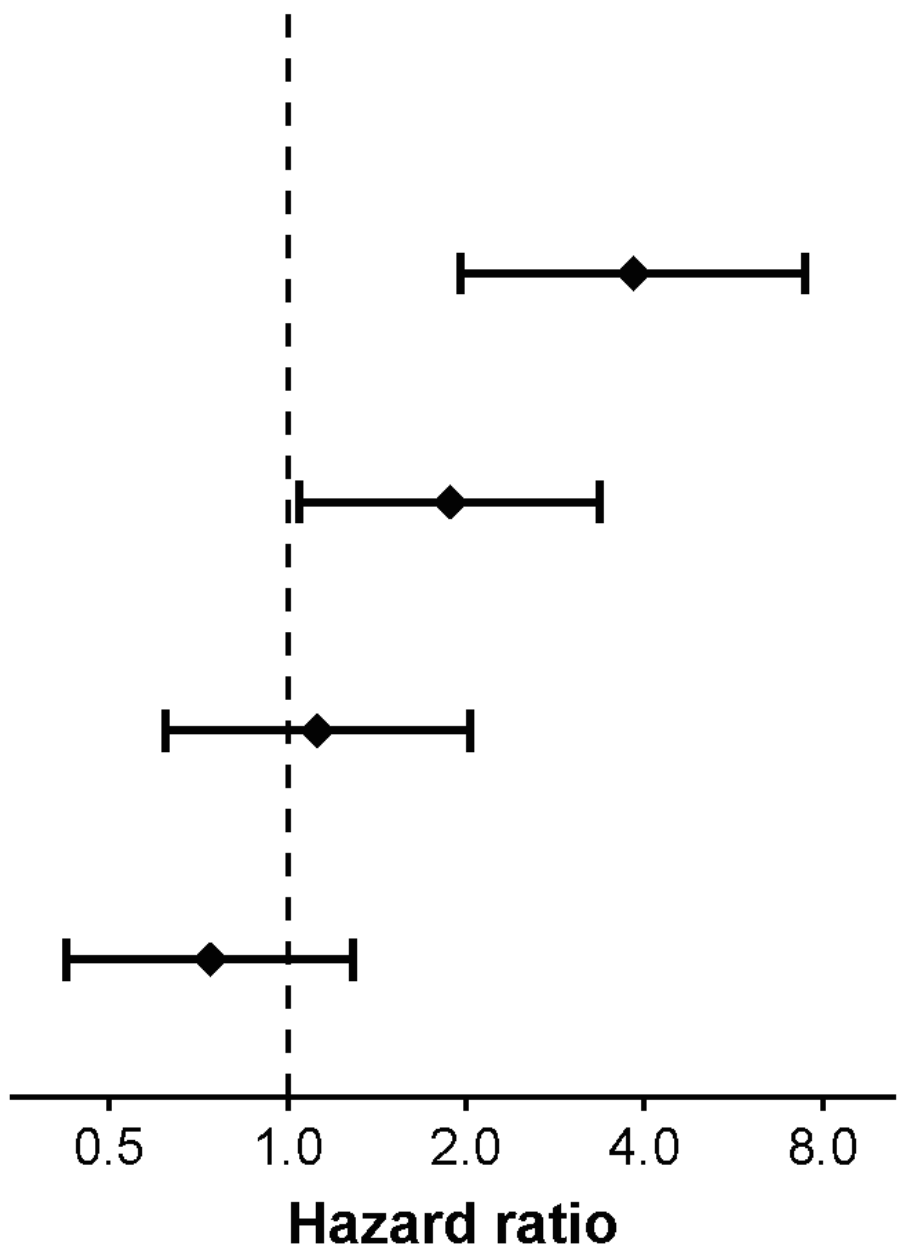

Supplement: Supplementary file 1 [file DataSheet1.pdf]
