# Supplementary material for: ORC6 marks a replication-active malignant epithelial state and is associated with immune-low features in oral squamous cell carcinoma
Source: Front Oncol. 2026 Jul 21;16:1882429. doi: 10.3389/fonc.2026.1882429 (PMC13433198; doi:10.3389/fonc.2026.1882429)
Supplement: Supplementary file 2 [file DataSheet2.pdf]

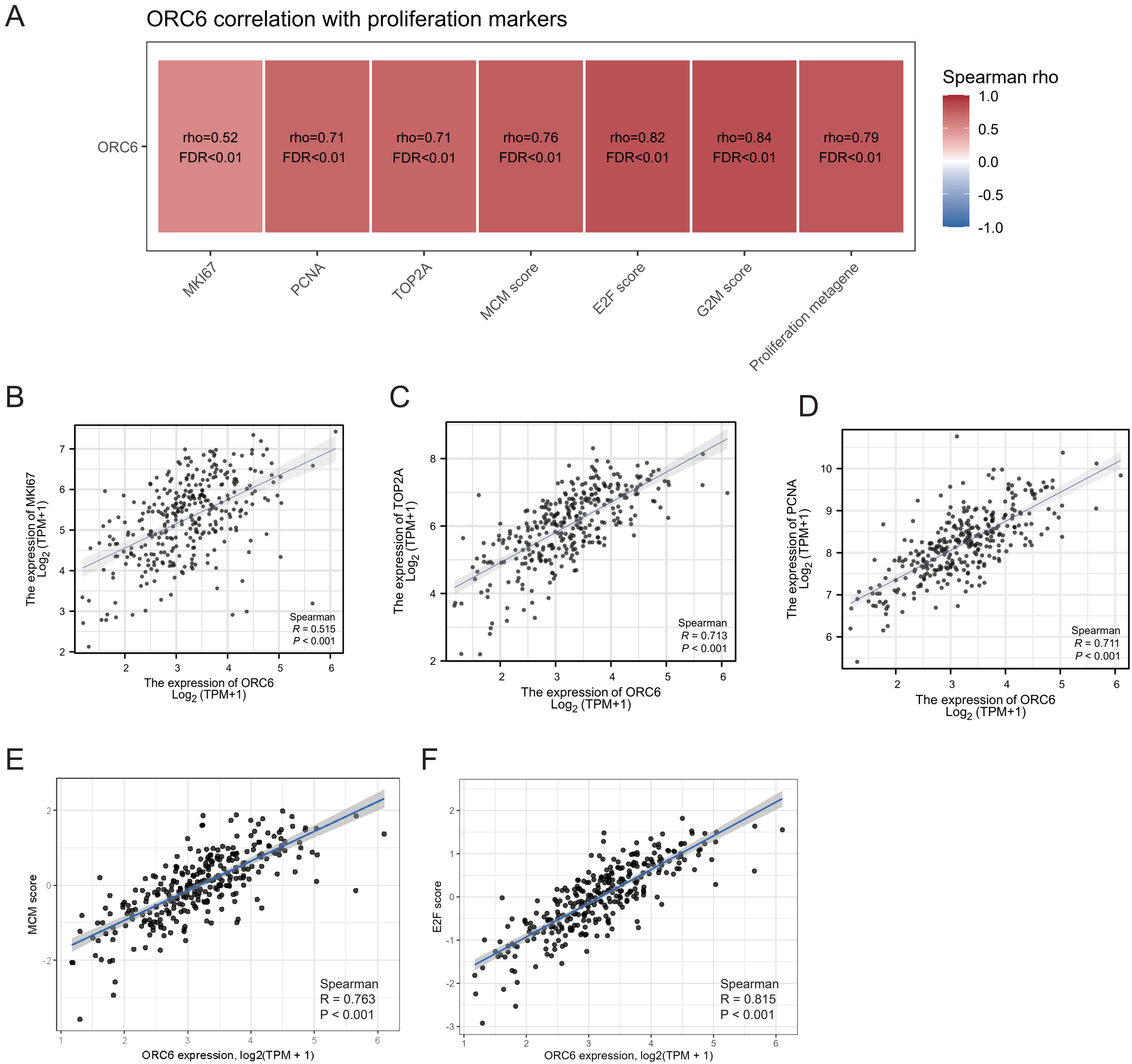
A
ORC6 correlation with proliferation markers
Spearman rho
1.0
0.5
0.0
-0.5
-1.0
ORC6
rho=0.52
FDR<0.01
rho=0.71
FDR<0.01
rho=0.71
FDR<0.01
rho=0.76
FDR<0.01
rho=0.82
FDR<0.01
rho=0.84
FDR<0.01
rho=0.79
FDR<0.01
MKI67
PCNA
TOP2A
MCM score
E2F score
G2M score
Proliferation metagene
B
The expression of MKI67 Log2 (TPM+1)
The expression of ORC6 Log2 (TPM+1)
Spearman
R = 0.515
P < 0.001
C
The expression of TOP2A Log2 (TPM+1)
The expression of ORC6 Log2 (TPM+1)
Spearman
R = 0.713
P < 0.001
D
The expression of PCNA Log2 (TPM+1)
The expression of ORC6 Log2 (TPM+1)
Spearman
R = 0.711
P < 0.001
E
MCM score
ORC6 expression, log2(TPM + 1)
Spearman
R = 0.763
P < 0.001
F
E2F score
ORC6 expression, log2(TPM + 1)
Spearman
R = 0.815
P < 0.001
