# Supplementary material for: ORC6 marks a replication-active malignant epithelial state and is associated with immune-low features in oral squamous cell carcinoma
Source: Front Oncol. 2026 Jul 21;16:1882429. doi: 10.3389/fonc.2026.1882429 (PMC13433198; doi:10.3389/fonc.2026.1882429)
Supplement: Supplementary file 3 [file DataSheet3.pdf]

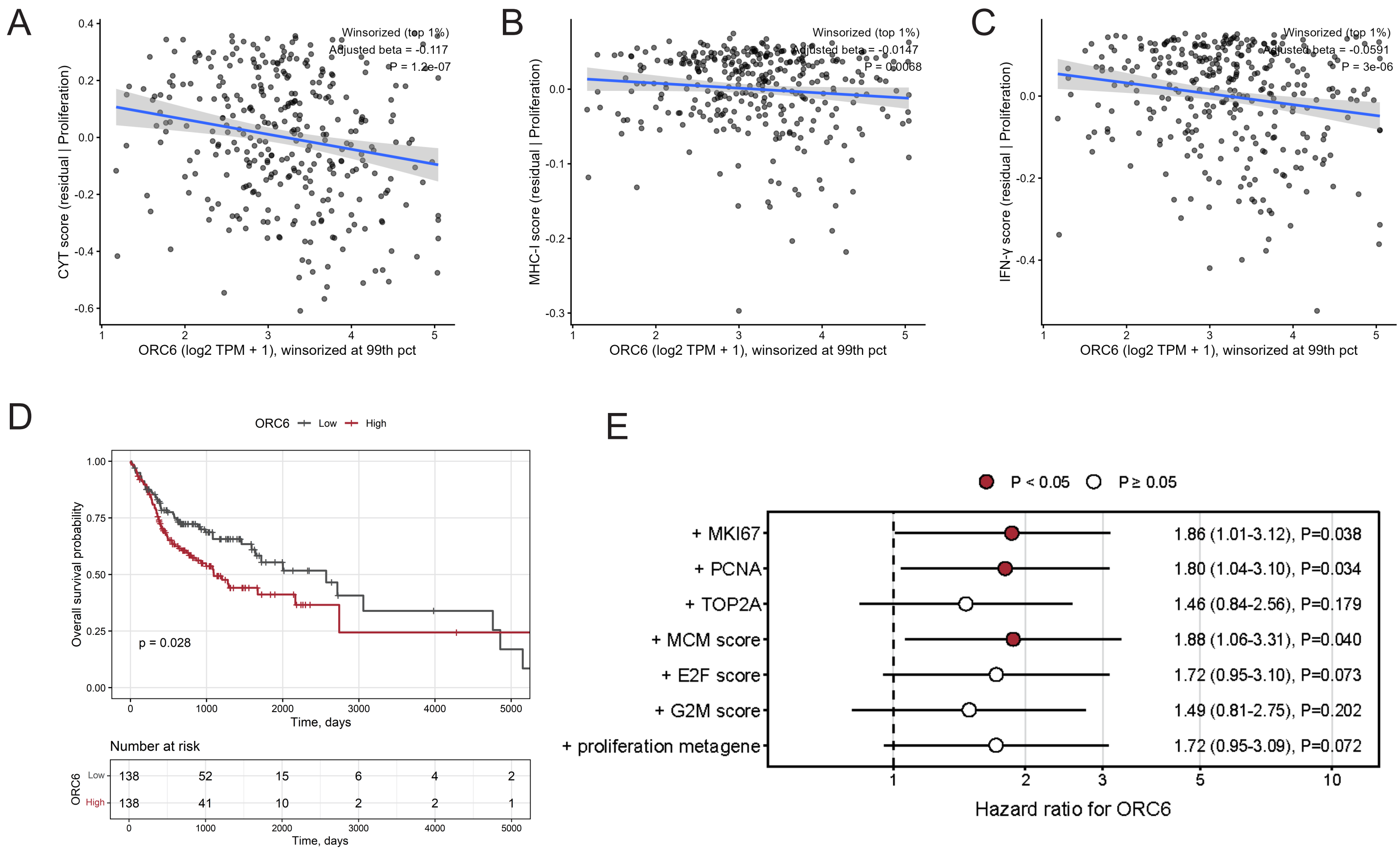

A
Winsorized (top 1%)
Adjusted beta = -0.117
P = 1.2e-07
CYT score (residual | Proliferation)
ORC6 (log2 TPM + 1), winsorized at 99th pct
B
Winsorized (top 1%)
Adjusted beta = -0.0147
P = 0.0068
MHC-I score (residual | Proliferation)
ORC6 (log2 TPM + 1), winsorized at 99th pct
C
Winsorized (top 1%)
Adjusted beta = -0.0591
P = 3e-06
IFN-γ score (residual | Proliferation)
ORC6 (log2 TPM + 1), winsorized at 99th pct
D
ORC6 Low High
Overall survival probability
p = 0.028
Time, days
Number at risk
ORC6
Low 138 52 15 6 4 2
High 138 41 10 2 2 1
Time, days
E
P < 0.05 P ≥ 0.05
+ MKI67 1.86 (1.01-3.12), P=0.038
+ PCNA 1.80 (1.04-3.10), P=0.034
+ TOP2A 1.46 (0.84-2.56), P=0.179
+ MCM score 1.88 (1.06-3.31), P=0.040
+ E2F score 1.72 (0.95-3.10), P=0.073
+ G2M score 1.49 (0.81-2.75), P=0.202
+ proliferation metagene 1.72 (0.95-3.09), P=0.072
Hazard ratio for ORC6
