# Supplementary material for: ORC6 marks a replication-active malignant epithelial state and is associated with immune-low features in oral squamous cell carcinoma
Source: Front Oncol. 2026 Jul 21;16:1882429. doi: 10.3389/fonc.2026.1882429 (PMC13433198; doi:10.3389/fonc.2026.1882429)
Supplement: Supplementary file 4 [file DataSheet4.pdf]

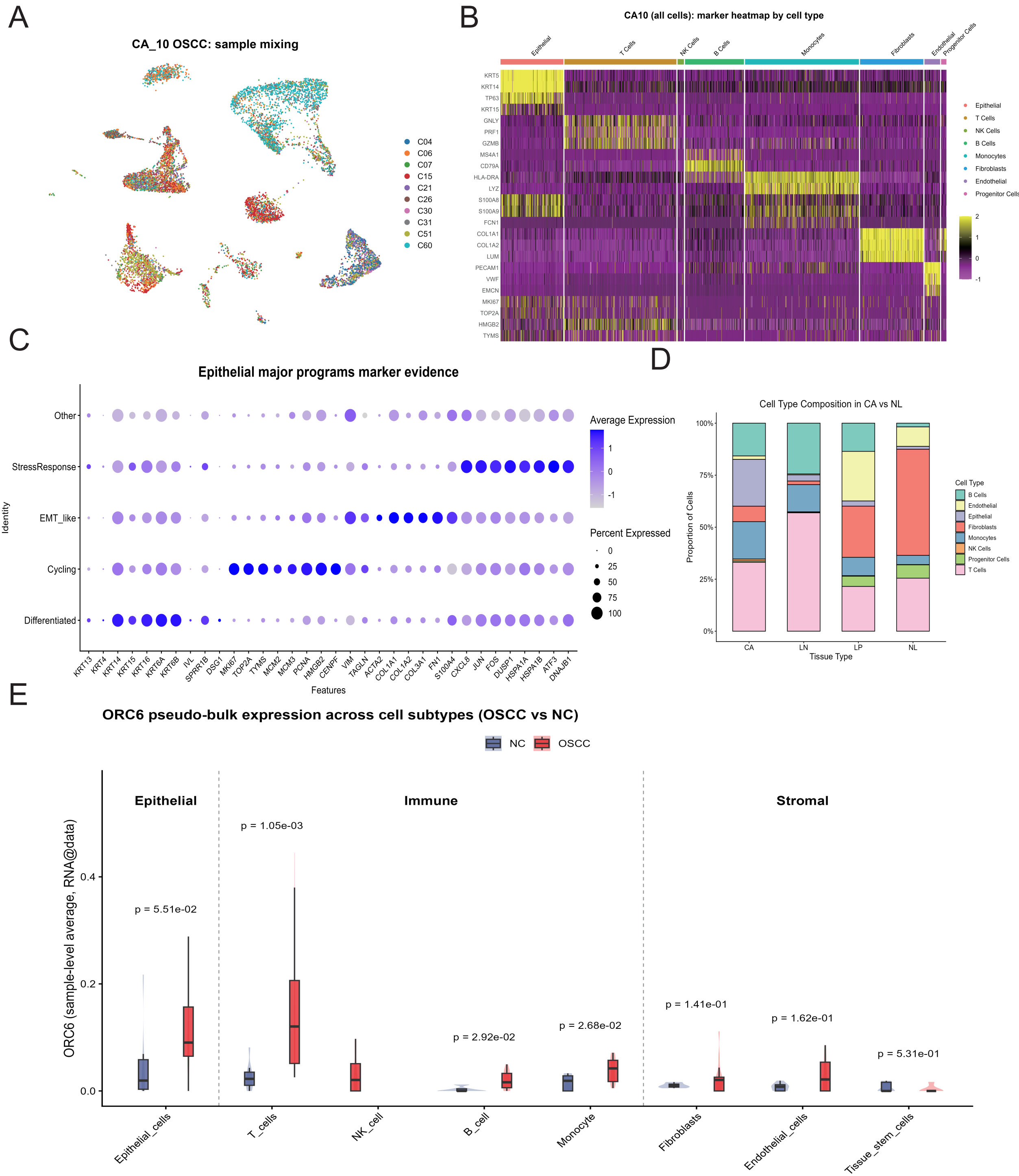
A
CA_10 OSCC: sample mixing
C04
C06
C07
C15
C21
C26
C30
C31
C51
C60
B
CA10 (all cells): marker heatmap by cell type
Epithelial
T Cells
NK Cells
B Cells
Monocytes
Fibroblasts
Endothelial
Progenitor Cells
KRT5
KRT14
TP63
KRT15
GNLY
PRF1
GZMB
MS4A1
CD79A
HLA-DRA
LYZ
S100A8
S100A9
FCN1
COL1A1
COL1A2
LUM
PECAM1
VWF
EMCN
MKI67
TOP2A
HMGB2
TYMS
C
Epithelial major programs marker evidence
Other
StressResponse
EMT_like
Cycling
Differentiated
Identity
KRT13 KRT4 KRT14 KRT15 KRT16 KRT6A KRT6B IVL SPRR1B DSG1 MKI67 TOP2A TYMS MCM2 MCM3 PCNA HMGB2 CENPF VIM TAGLN ACTA2 COL1A1 COL1A2 COL3A1 FN1 S100A4 CXCL8 JUN FOS DUSP1 HSPA1A HSPA1B ATF3 DNAJB1
Features
Average Expression
Percent Expressed
0
25
50
75
100
D
Cell Type Composition in CA vs NL
Proportion of Cells
100%
75%
50%
25%
0%
CA
LN
LP
NL
Tissue Type
Cell Type
B Cells
Endothelial
Epithelial
Fibroblasts
Monocytes
NK Cells
Progenitor Cells
T Cells
E
ORC6 pseudo-bulk expression across cell subtypes (OSCC vs NC)
NC
OSCC
ORC6 (sample-level average, RNA@data)
Epithelial
Immune
Stromal
p = 5.51e-02
p = 1.05e-03
p = 2.92e-02
p = 2.68e-02
p = 1.41e-01
p = 1.62e-01
p = 5.31e-01
0.0
0.2
0.4
Epithelial_cells
T_cells
NK_cell
B_cell
Monocyte
Fibroblasts
Endothelial_cells
Tissue_stem_cells
