# Supplementary material for: ORC6 marks a replication-active malignant epithelial state and is associated with immune-low features in oral squamous cell carcinoma
Source: Front Oncol. 2026 Jul 21;16:1882429. doi: 10.3389/fonc.2026.1882429 (PMC13433198; doi:10.3389/fonc.2026.1882429)

Regression efficacy: absolute gene–cell-cycle correlation decreases in residual space

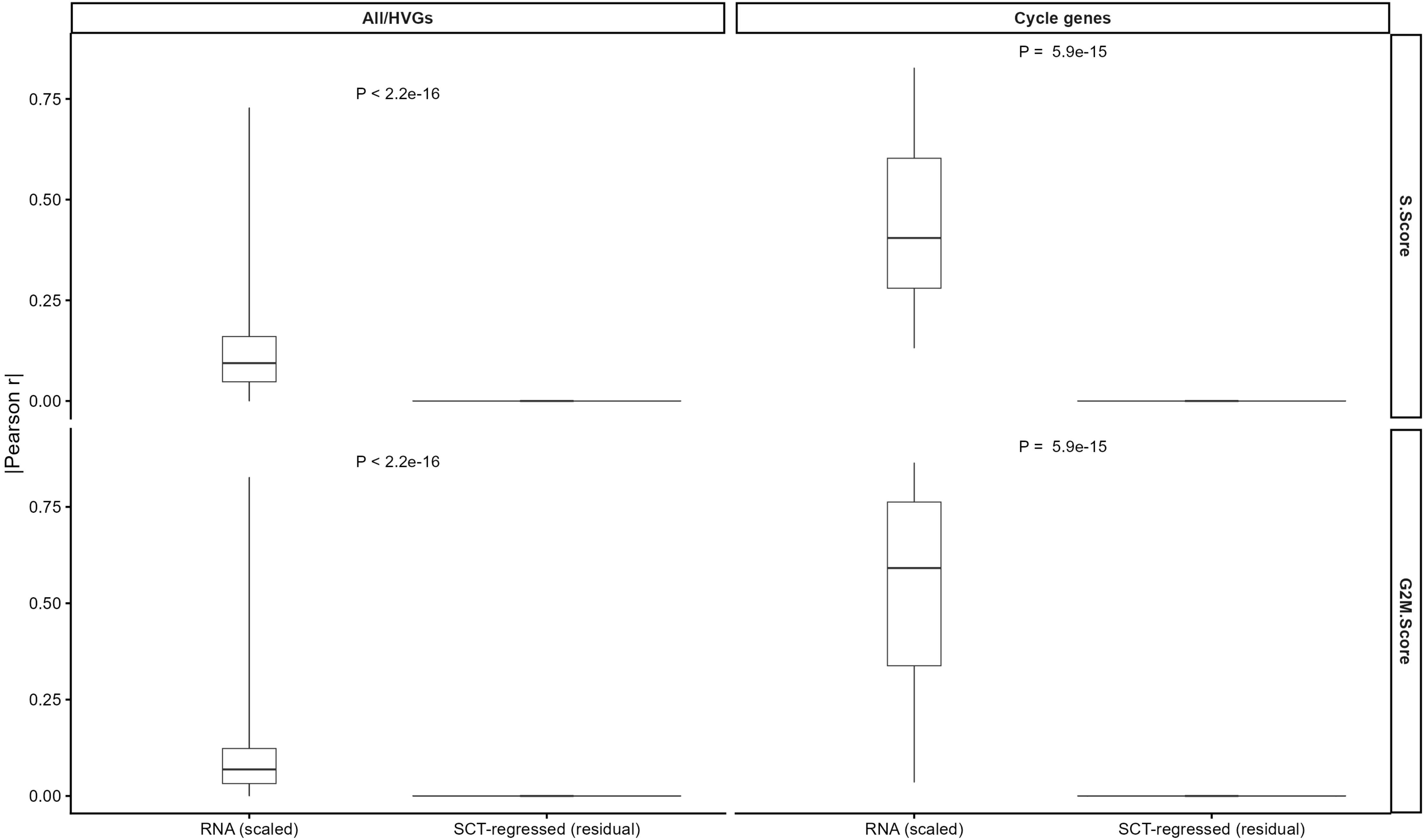

Supplement: Supplementary file 5 [file DataSheet5.pdf]
