# Supplementary material for: ORC6 marks a replication-active malignant epithelial state and is associated with immune-low features in oral squamous cell carcinoma
Source: Front Oncol. 2026 Jul 21;16:1882429. doi: 10.3389/fonc.2026.1882429 (PMC13433198; doi:10.3389/fonc.2026.1882429)
Supplement: Supplementary file 6 [file DataSheet6.pdf]

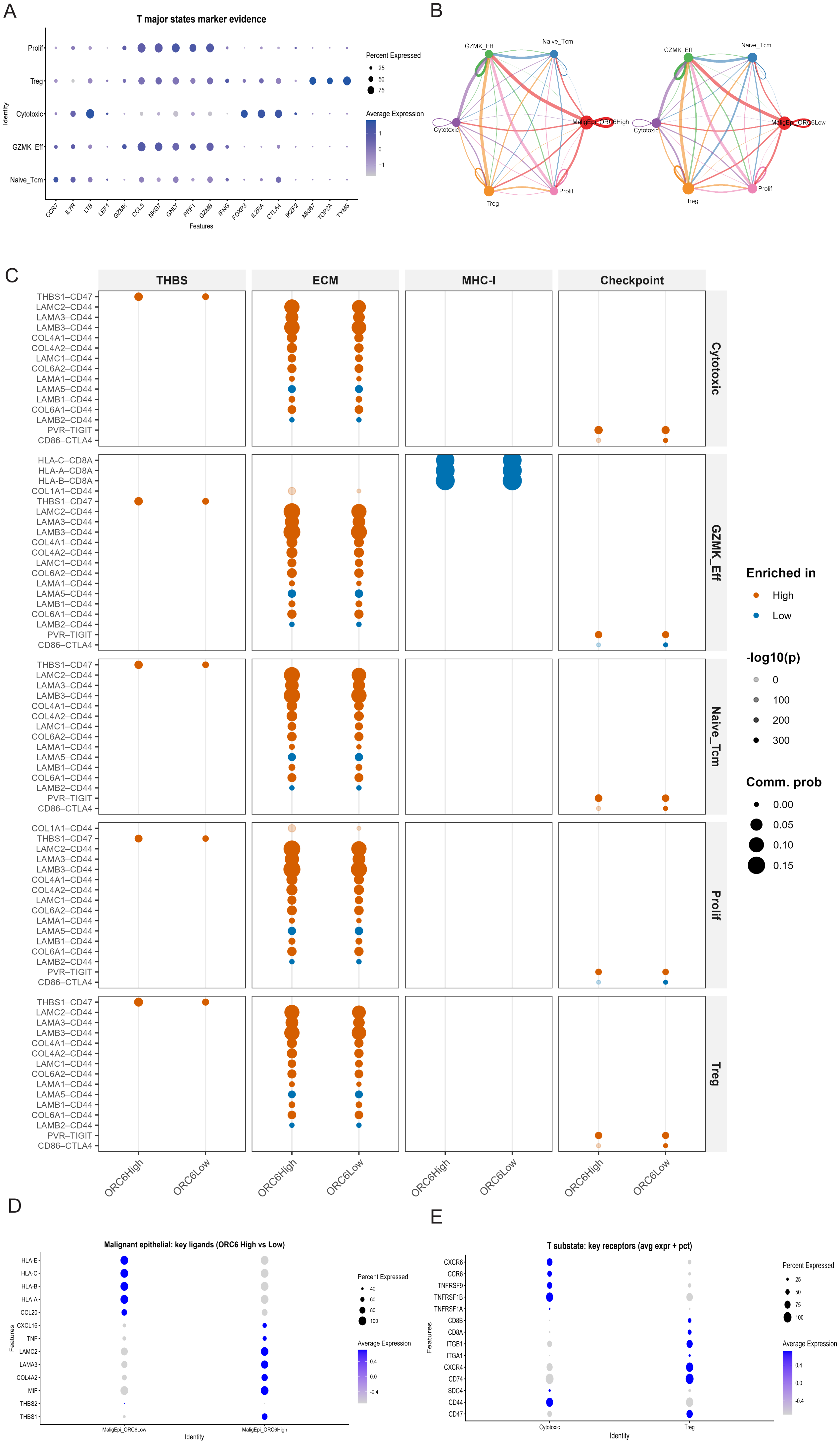
A
T major states marker evidence
Prolif
Treg
Cytotoxic
GZMK_Eff
Naive_Tcm
Identity
CCR7 IL7R LTB LEF1 GZMK CCL5 NKG7 GNLY PRF1 GZMB IFNG FOXP3 IL2RA CTLA4 IKZF2 MKI67 TOP2A TYMS
Features
Percent Expressed
25
50
75
Average Expression
1
0
−1
B
GZMK_Eff
Naive_Tcm
Cytotoxic
MaligEpi_ORC6High
Treg
Prolif
GZMK_Eff
Naive_Tcm
Cytotoxic
MaligEpi_ORC6Low
Treg
Prolif
C
THBS
ECM
MHC-I
Checkpoint
Cytotoxic
GZMK_Eff
Naive_Tcm
Prolif
Treg
THBS1–CD47
LAMC2–CD44
LAMA3–CD44
LAMB3–CD44
COL4A1–CD44
COL4A2–CD44
LAMC1–CD44
COL6A2–CD44
LAMA1–CD44
LAMA5–CD44
LAMB1–CD44
COL6A1–CD44
LAMB2–CD44
PVR–TIGIT
CD86–CTLA4
HLA-C–CD8A
HLA-A–CD8A
HLA-B–CD8A
COL1A1–CD44
ORC6High
ORC6Low
Enriched in
High
Low
-log10(p)
0
100
200
300
Comm. prob
0.00
0.05
0.10
0.15
D
Malignant epithelial: key ligands (ORC6 High vs Low)
HLA-E
HLA-C
HLA-B
HLA-A
CCL20
CXCL16
TNF
LAMC2
LAMA3
COL4A2
MIF
THBS2
THBS1
Features
MaligEpi_ORC6Low
MaligEpi_ORC6High
Identity
Percent Expressed
40
60
80
100
Average Expression
0.4
0.0
-0.4
E
T substate: key receptors (avg expr + pct)
CXCR6
CCR6
TNFRSF9
TNFRSF1B
TNFRSF1A
CD8B
CD8A
ITGB1
ITGA1
CXCR4
CD74
SDC4
CD44
CD47
Features
Cytotoxic
Treg
Identity
Percent Expressed
25
50
75
100
Average Expression
0.4
0.0
-0.4
