# Supplementary material for: ORC6 marks a replication-active malignant epithelial state and is associated with immune-low features in oral squamous cell carcinoma
Source: Front Oncol. 2026 Jul 21;16:1882429. doi: 10.3389/fonc.2026.1882429 (PMC13433198; doi:10.3389/fonc.2026.1882429)
Supplement: Supplementary file 7 [file DataSheet7.pdf]

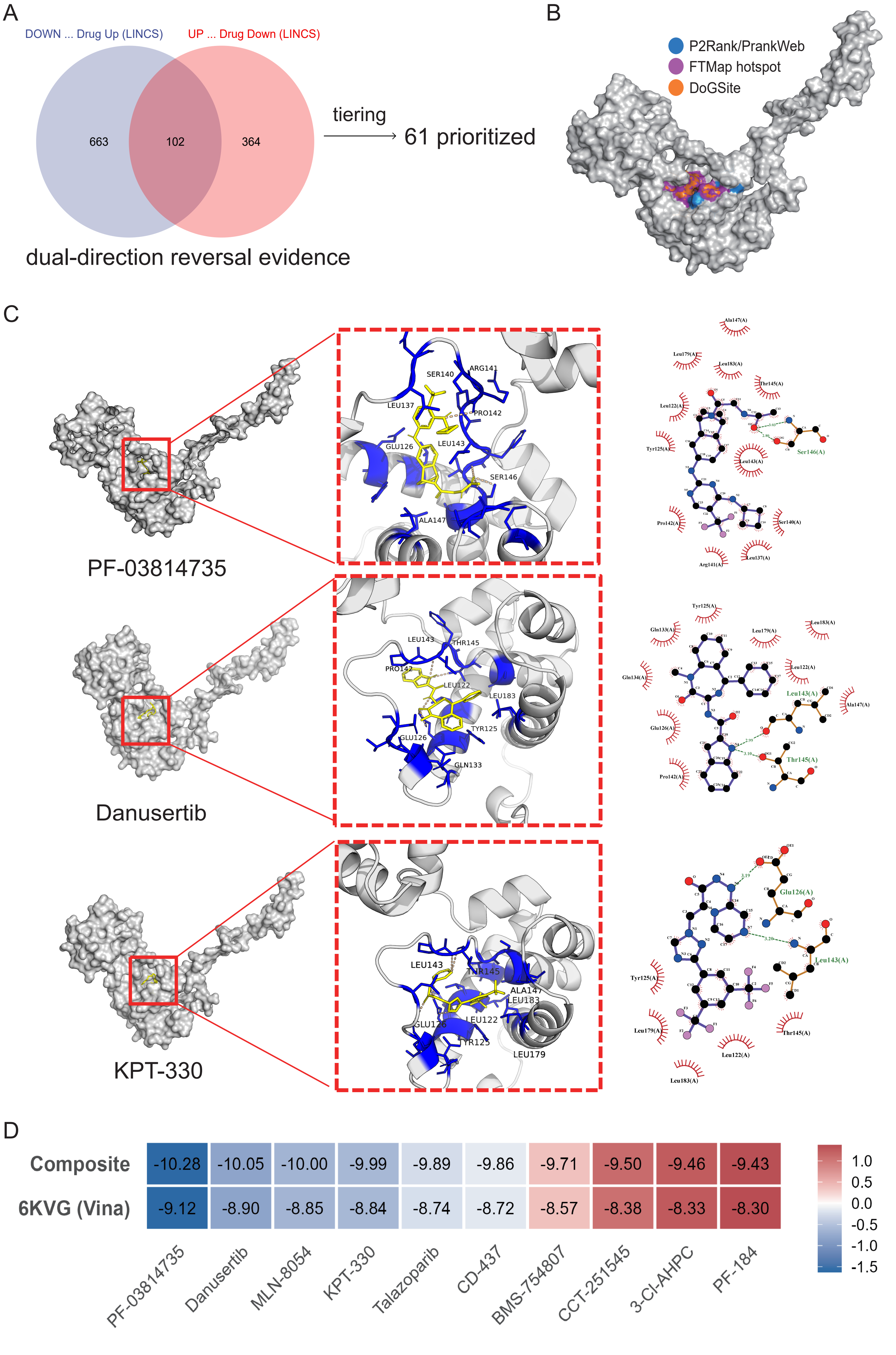
A
DOWN ... Drug Up (LINCS)
UP ... Drug Down (LINCS)
663
102
364
tiering
61 prioritized
dual-direction reversal evidence
B
P2Rank/PrankWeb
FTMap hotspot
DoGSite
C
SER140
ARG141
LEU137
PRO142
GLU126
LEU143
SER146
ALA147
PF-03814735
LEU143
THR145
PRO142
LEU122
LEU183
TYR125
GLU126
GLN133
Danusertib
LEU143
THR145
ALA147
LEU183
LEU122
GLU126
TYR125
LEU179
KPT-330
D
Composite
6KVG (Vina)
-10.28
-10.05
-10.00
-9.99
-9.89
-9.86
-9.71
-9.50
-9.46
-9.43
-9.12
-8.90
-8.85
-8.84
-8.74
-8.72
-8.57
-8.38
-8.33
-8.30
PF-03814735
Danusertib
MLN-8054
KPT-330
Talazoparib
CD-437
BMS-754807
CCT-251545
3-Cl-AHPC
PF-184
1.0
0.5
0.0
-0.5
-1.0
-1.5
