# Supplementary material for: ORC6 marks a replication-active malignant epithelial state and is associated with immune-low features in oral squamous cell carcinoma
Source: Front Oncol. 2026 Jul 21;16:1882429. doi: 10.3389/fonc.2026.1882429 (PMC13433198; doi:10.3389/fonc.2026.1882429)
Supplement: Supplementary file 8 [file DataSheet8.pdf]

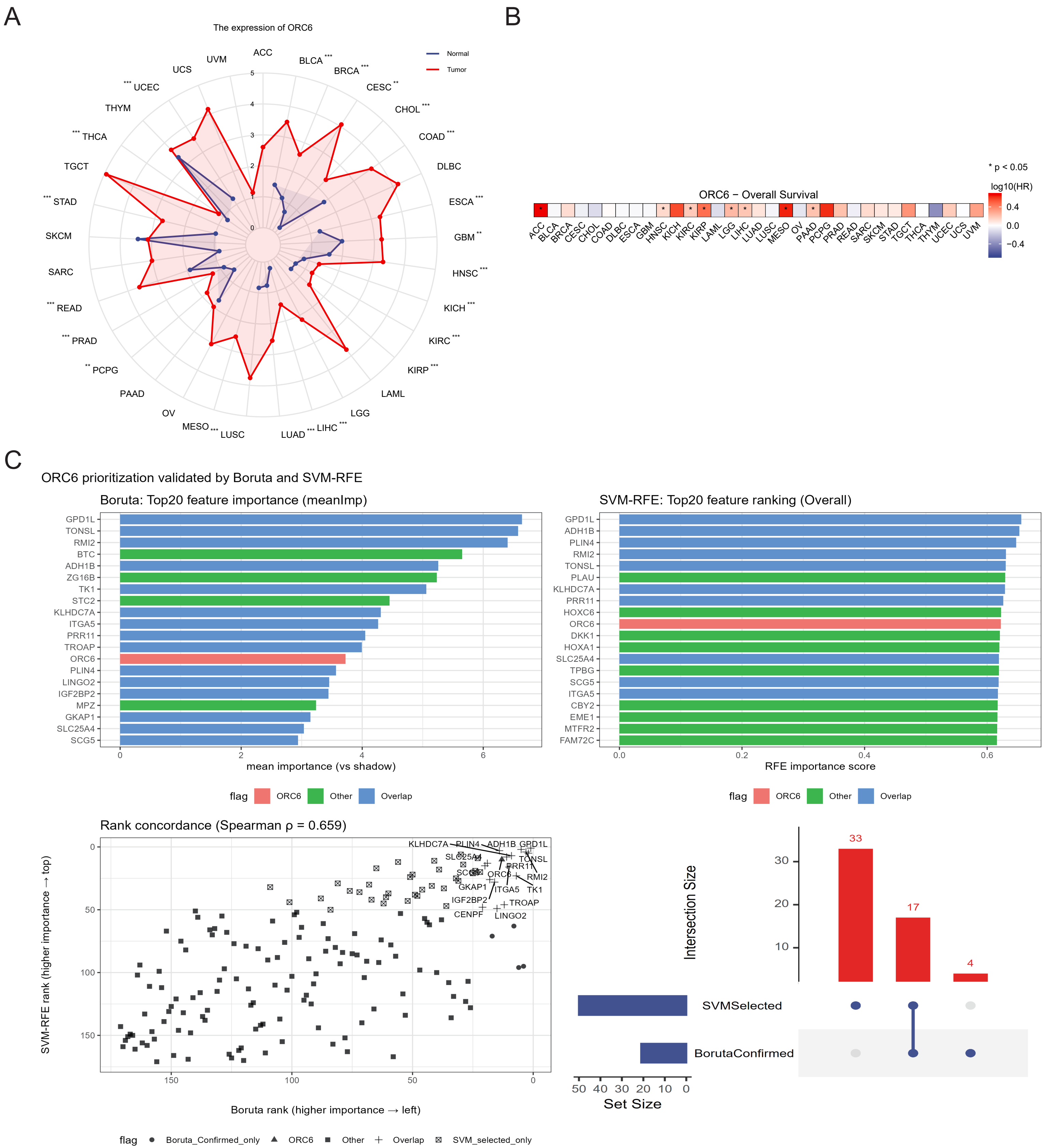

A
The expression of ORC6
Normal
Tumor
B
ORC6 − Overall Survival
* p < 0.05
log10(HR)
C
ORC6 prioritization validated by Boruta and SVM-RFE
Boruta: Top20 feature importance (meanImp)
mean importance (vs shadow)
SVM-RFE: Top20 feature ranking (Overall)
RFE importance score
flag ORC6 Other Overlap
Rank concordance (Spearman ρ = 0.659)
SVM-RFE rank (higher importance → top)
Boruta rank (higher importance → left)
flag Boruta_Confirmed_only ORC6 Other Overlap SVM_selected_only
Intersection Size
SVMSelected
BorutaConfirmed
Set Size
