# Supplementary material for: ORC6 marks a replication-active malignant epithelial state and is associated with immune-low features in oral squamous cell carcinoma
Source: Front Oncol. 2026 Jul 21;16:1882429. doi: 10.3389/fonc.2026.1882429 (PMC13433198; doi:10.3389/fonc.2026.1882429)
Supplement: Supplementary file 9 [file DataSheet9.pdf]

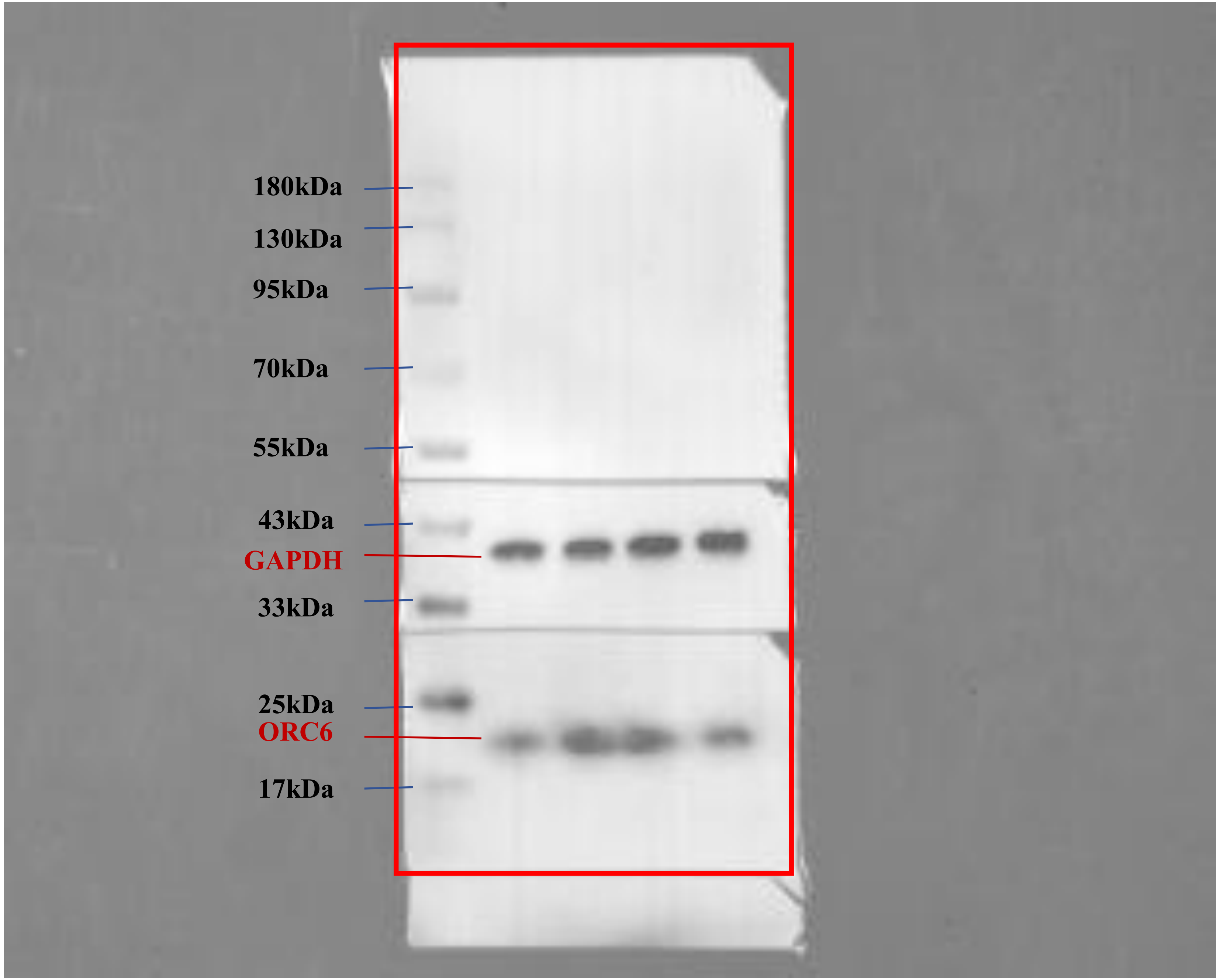


12.5% Tris-Glycine SDS-PAGE

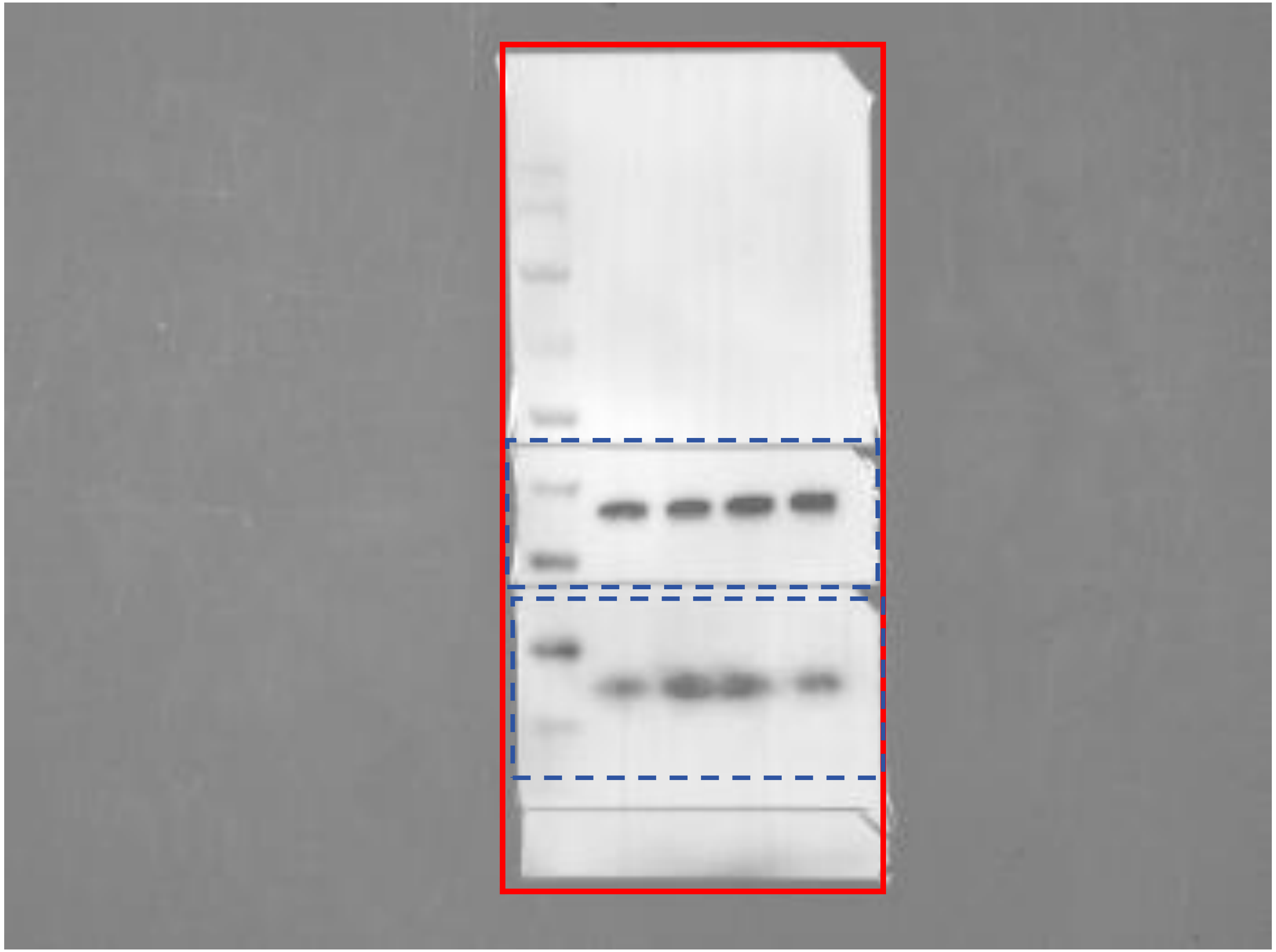

**Full original images of the membrane segments**

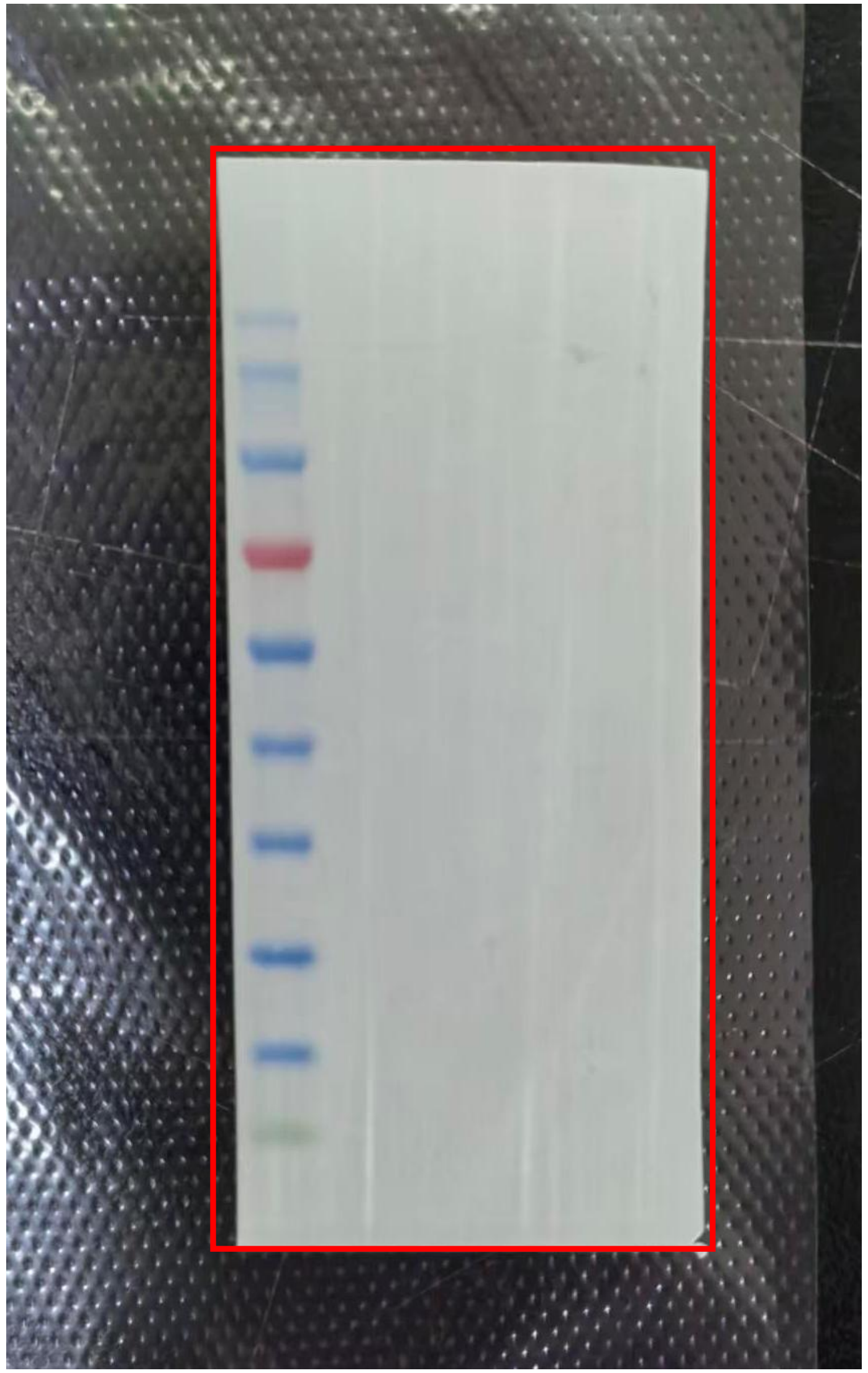

Before cutting

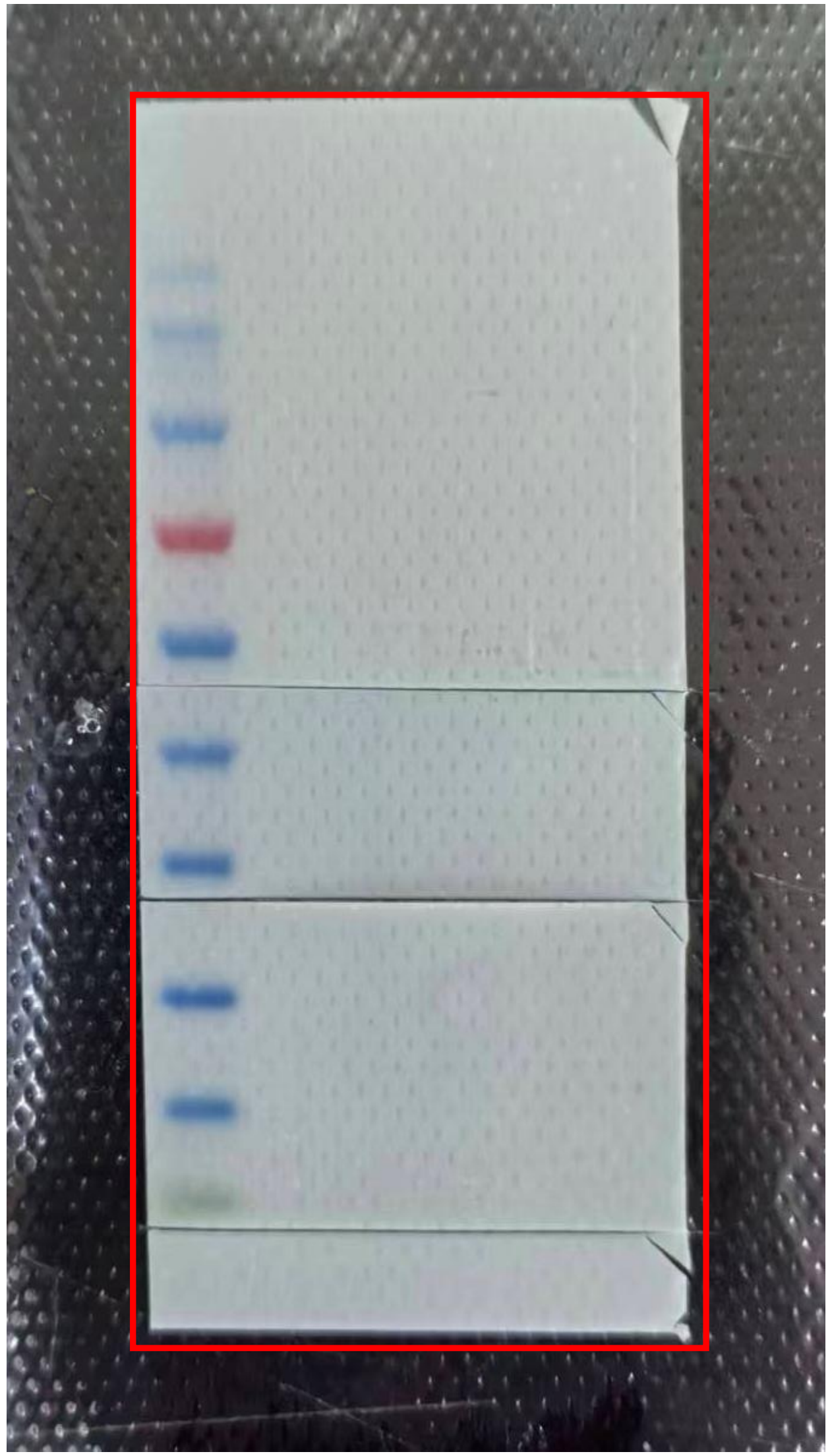

Membrane after physical cutting and before primary-antibody incubation

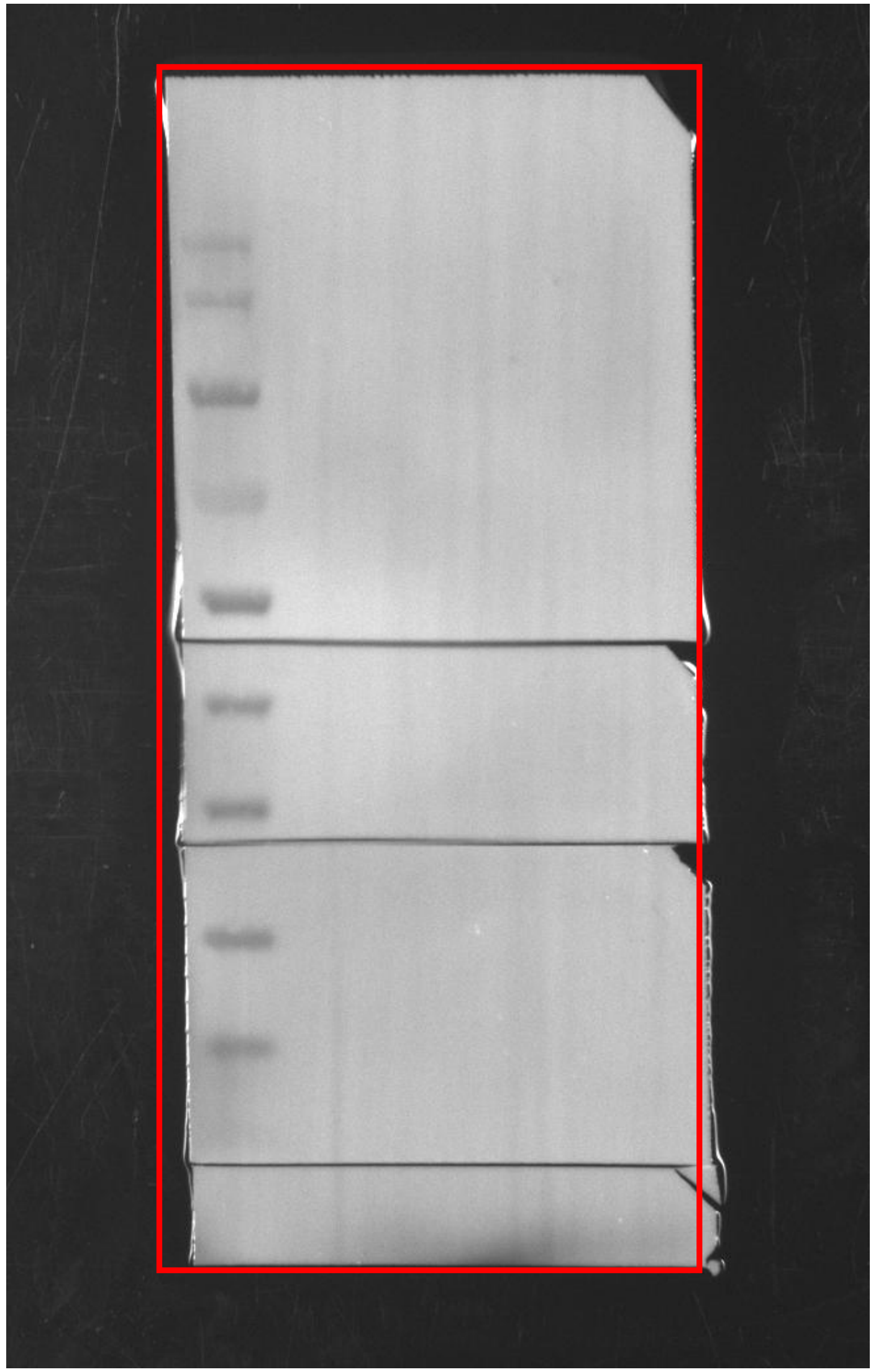

Developed marker image of the membrane segments

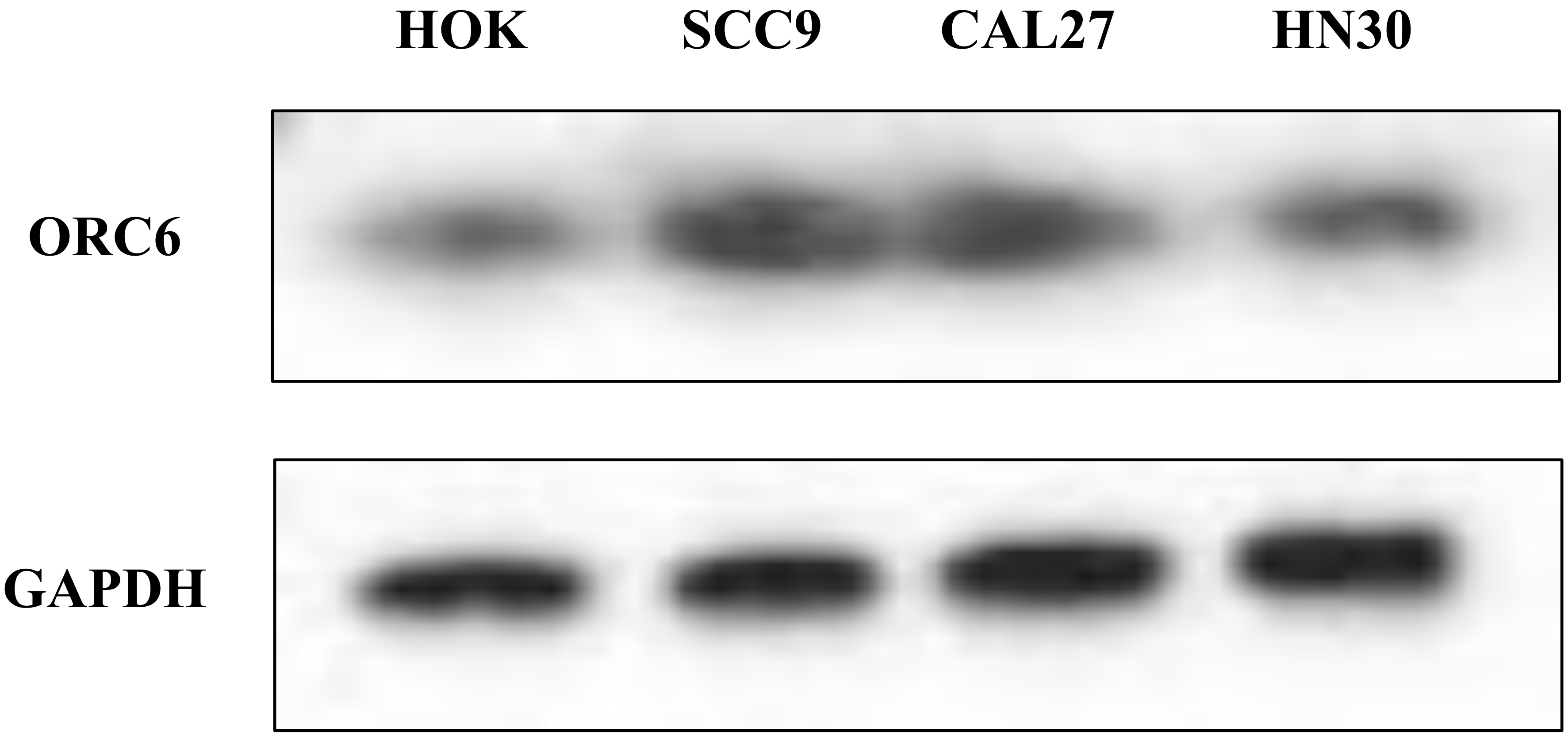
HOK
SCC9
CAL27
HN30
ORC6
GAPDH
