# Supplementary material for: ORC6 marks a replication-active malignant epithelial state and is associated with immune-low features in oral squamous cell carcinoma
Source: Front Oncol. 2026 Jul 21;16:1882429. doi: 10.3389/fonc.2026.1882429 (PMC13433198; doi:10.3389/fonc.2026.1882429)
Supplement: Supplementary file 10 [file SupplementaryFile1.docx]

**Supplementary Methods**

1. **Construction of proliferation-related covariates and proliferation-adjusted regression analyses**

**1.1 Construction of proliferation-related covariates**

To assess whether the prognostic and immune-related associations of ORC6 were influenced by canonical proliferation programs, several proliferation-related covariates were constructed at the bulk transcriptomic level. Single-gene proliferation markers included MKI67, PCNA, and TOP2A. In addition, an MCM score was calculated using MCM2, MCM3, MCM4, MCM5, MCM6, and MCM7.

Representative E2F and G2/M scores were calculated using curated canonical cell-cycle gene sets. The E2F representative score included E2F1, E2F2, E2F3, E2F7, E2F8, TFDP1, TFDP2, MCM2, MCM3, MCM4, MCM5, MCM6, MCM7, MCM10, PCNA, TYMS, DHFR, RRM1, RRM2, CDC6, CDT1, CDC25A, CCNE1, CCNE2, CDK2, BRCA1, BRCA2, RAD51, FEN1, RFC2, RFC3, RFC4, and RFC5. The G2/M representative score included CCNB1, CCNB2, CDK1, CDC20, CDC25C, AURKA, AURKB, BUB1, BUB1B, BIRC5, PLK1, TOP2A, CENPA, CENPE, CENPF, MKI67, NUSAP1, TPX2, KIF2C, KIF11, KIF20A, KIF23, MAD2L1, TTK, UBE2C, UBE2S, MELK, and FOXM1. A proliferation metagene score was further calculated using MKI67, PCNA, TOP2A, MCM2, MCM3, MCM4, MCM5, MCM6, MCM7, CCNA2, CCNB1, CCNB2, CDK1, CDC20, CENPF, BIRC5, and UBE2C.

For all multigene scores, the expression value of each gene was first standardized across samples using z-score transformation. The final score was then calculated as the arithmetic mean of the z-scored expression values of all available genes within the corresponding gene set. This approach allowed different proliferation-related programs to be represented as sample-level continuous covariates while reducing the influence of individual gene scale differences.

**1.2 Proliferation-adjusted immune association models**

To evaluate whether the association between ORC6 and immune-related transcriptional features remained detectable after accounting for canonical proliferation programs, multivariable linear regression models were fitted for representative immune outcomes. The immune outcomes included cytolytic activity score, IFN-γ response score, and MHC-I/antigen-presentation score. Each proliferation-related variable was incorporated separately to avoid excessive collinearity among proliferation covariates.

The general model form was:

immune outcome ~ ORC6 + proliferation covariate

where ORC6 was modeled as a continuous expression variable and the proliferation covariate represented MKI67, PCNA, TOP2A, MCM score, E2F score, G2/M score, or the proliferation metagene score. The adjusted regression coefficient of ORC6 was extracted from each model to evaluate the direction and robustness of the ORC6-associated immune relationship after proliferation adjustment.

**1.3 Clinical- and proliferation-adjusted Cox sensitivity analyses**

To assess whether the prognostic association of ORC6 was influenced by proliferation-related programs, proliferation-adjusted Cox proportional hazards sensitivity models were fitted for overall survival. ORC6 was analyzed using median-based high versus low stratification, with the ORC6-low group used as the reference. Each proliferation-related covariate was incorporated separately into the Cox model.

The general model form was:

Surv(OS time, OS status) ~ ORC6 group + pathologic T stage + pathologic N stage + age + lymph node neck dissection + proliferation covariate

where the proliferation covariate represented MKI67, PCNA, TOP2A, MCM score, E2F score, G2/M score, or the proliferation metagene score. Hazard ratios and 95% confidence intervals for ORC6-high versus ORC6-low tumors were extracted from each model.

**1.4 Interpretation of proliferation-adjusted analyses**

These proliferation-adjusted models were designed as sensitivity analyses rather than as definitive proof of proliferation-independent prognostic or immune-regulatory effects. Because ORC6 is intrinsically involved in DNA replication licensing and cell-cycle progression, the adjusted analyses were interpreted to determine whether ORC6-associated immune and survival patterns remained directionally consistent after accounting for canonical proliferation-related covariates. Associations that were attenuated after adjustment were interpreted as evidence that ORC6-related clinical or immune features may be partially linked to the broader replication-active tumor state.

**2. Exploratory reversal-signature and structure-based compound prioritization**

### 2.1 Reversal-guided compound prioritization

Differential gene signatures from ORC6-high versus ORC6-low malignant epithelial cells were separated into upregulated and downregulated components. Direction-constrained reversal screening was then performed using Enrichr-based chemical perturbation enrichment against the LINCS L1000 Chem Pert Consensus Sigs library. Because entries in this library carry direction labels (“Drug Up” or “Drug Down”), reversal-oriented filtering was applied to identify compounds predicted to oppose the ORC6-high state.

For the ORC6-high upregulated signature, only enriched terms containing “Down” in the entry name were retained. For the ORC6-high downregulated signature, only enriched terms containing “Up” were retained. In both analyses, adjusted P < 0.01 was used as the selection threshold. The two filtered result sets were then intersected by drug name to obtain candidate compounds supported by bidirectional reversal evidence. Candidate ranking was based on adjusted significance from the enrichment results.

**2.2 Filtering of broadly cytotoxic compounds**

Because the ORC6-high state is strongly linked to replication and cell-cycle programs, reversal screening can preferentially enrich for broadly cytotoxic agents. After excluding entries for which chemical structure information could not be clearly retrieved from PubChem or ChEMBL, compounds with typical antimitotic, microtubule-toxic, or otherwise nonspecific cytotoxic properties were annotated as broadly cytotoxic and deprioritized in the final interpretation.

**2.3 ORC6 structure preparation and pocket definition**

The PDB structure 6KVG was used as the structural source for ORC6, and chain A was consistently used as the receptor for site prediction, hotspot mapping, and molecular docking. A consensus binding pocket was defined by integrating predictions from DoGSite, P2Rank, and FTMap. Docking grids were generated using a residue-defined boxing strategy based on the consensus pocket. Representative residues used to define the docking box included A121, A122, A139–A146, and A161–A167. Receptor preprocessing included removal of crystallographic water molecules and heteroatoms, residue-name standardization, protonation and hydrogen addition, and structural optimization according to the docking workflow.

### 2.4 Molecular docking and post-docking rescoring

Molecular docking was performed using AutoDock Vina (v1.2.3). For each ligand, the conformation with the lowest predicted binding energy was selected as the representative pose. Multiple scoring functions were integrated for rescoring, including Vina score, X-Score, ChemScore, and GlideScore, and a composite ranking score was calculated.

### 2.5 ADMET and drug-likeness evaluation

ADMET properties and drug-likeness features were additionally evaluated to prioritize compounds with more favorable translational profiles.

**2.6 Interpretive boundaries**

### These analyses were exploratory and hypothesis-generating only. Reversal-signature analysis may preferentially identify broadly anti-proliferative compounds, and molecular docking does not establish target engagement or biological efficacy. Therefore, the candidate compounds nominated here should not be interpreted as validated ORC6-targeted agents or therapeutically actionable compounds.
