# Supplementary material for: ORC6 marks a replication-active malignant epithelial state and is associated with immune-low features in oral squamous cell carcinoma
Source: Front Oncol. 2026 Jul 21;16:1882429. doi: 10.3389/fonc.2026.1882429 (PMC13433198; doi:10.3389/fonc.2026.1882429)
Supplement: Supplementary file 11 [file SupplementaryFile2.docx]

**Table S1.** *Robustness of associations between ORC6 and immune-related signatures before and after winsorization of the top 1% of ORC6 expression values.*

| **Outcome** | **Beta (raw)** | **P value (raw)** | **FDR (raw)** | **Beta (winsorized)** | **P value (winsorized)** | **FDR (winsorized)** | **ORC6 q99 threshold (log2 TPM + 1)** |
| --- | --- | --- | --- | --- | --- | --- | --- |
| IFNG_Response | -0.057 | 3.55e-06 | 0.000 | -0.059 | 3.03e-06 | 0.000 | 5.04 |
| CYT | -0.111 | 2.30e-07 | 0.000 | -0.117 | 1.24e-07 | 0.000 | 5.04 |
| MHC_I | -0.014 | 0.008 | 0.008 | -0.015 | 0.007 | 0.007 | 5.04 |

**Note:** Raw and winsorized models are shown side by side to evaluate the robustness of the observed associations. Winsorization was performed at the 99th percentile (top 1%) of ORC6 expression values. Negative beta coefficients indicate inverse associations between ORC6 and the corresponding immune-related signature. Abbreviations: CYT, cytolytic activity; FDR, false discovery rate; IFNG, interferon-gamma.
