# Supplementary material for: ORC6 marks a replication-active malignant epithelial state and is associated with immune-low features in oral squamous cell carcinoma
Source: Front Oncol. 2026 Jul 21;16:1882429. doi: 10.3389/fonc.2026.1882429 (PMC13433198; doi:10.3389/fonc.2026.1882429)
Supplement: Supplementary file 12 [file SupplementaryFile3.docx]

**Table S2.Primer sequences and siRNA oligonucleotides used in this study**

All nucleotide sequences are listed in the 5′→3′ direction. siRNA duplexes are shown as sense and antisense strands with dT overhangs retained as originally designed.

**A. qRT-PCR primer sequences**

| **Target gene** | **Forward primer (5′→3′)** | **Reverse primer (5′→3′)** |
| --- | --- | --- |
| ORC6 | ACAAGGAGACATATCAGAGCTGT | AGTGGCCTGGATAAGTCAAGAT |
| GAPDH | CTGGGCTACACTGAGCACC | AAGTGGTCGTTGAGGGCAATG |
| TAP1 | GTGGCCGCAGTGGGACAAGAG | AGGGCACTGGTGGCATCATC |
| B2M | TGCTGTCTCCATGTTTGATGTATCT | TCTCTGCTCCCCACCTCTAAGT |
| HLA-A | TGTGGAGGAGGAAGAGCTCAGATA | ACAAGGCAGCTGTCTCACACTTTA |
| NLRC5 | CCGTGGTACTCACATTTGCC | CCTTCGAGATCTCTGGGACA |

**B. siRNA oligonucleotide sequences targeting ORC6**

| **Target** | **siRNA ID** | **Strand** | **Sequence (5′→3′)** |
| --- | --- | --- | --- |
| ORC6 | H23594-siORC6-1 | Sense | GUUUAGCUGUAUAGAAGCA(dT)(dT) |
| ORC6 | H23594-siORC6-1 | Antisense | UGCUUCUAUACAGCUAAAC(dT)(dT) |
| ORC6 | H23594-siORC6-2 | Sense | ACAGUUUAGCUGUAUAGAA(dT)(dT) |
| ORC6 | H23594-siORC6-2 | Antisense | UUCUAUACAGCUAAACUGU(dT)(dT) |

Abbreviations: GAPDH, glyceraldehyde-3-phosphate dehydrogenase; siRNA, small interfering RNA.
