# Supplementary material for: ORC6 marks a replication-active malignant epithelial state and is associated with immune-low features in oral squamous cell carcinoma
Source: Front Oncol. 2026 Jul 21;16:1882429. doi: 10.3389/fonc.2026.1882429 (PMC13433198; doi:10.3389/fonc.2026.1882429)
Supplement: Supplementary file 13 [file SupplementaryFile4.docx]

**Table S3. ADMET-related properties of candidate compounds.**

*Summary of physicochemical and drug-likeness descriptors for candidate compounds included in the prioritization analysis.*

| **Compound** | **MW (Da)** | **LogP** | **TPSA (Å²)** | **HBD** | **HBA** | **Rotatable bonds** | **Heavy atoms** | **Drug-like** |
| --- | --- | --- | --- | --- | --- | --- | --- | --- |
| PF-03814735 | 474.487 | 4.0577 | 99.25 | 3 | 6 | 6 | 34 | Yes |
| Danusertib | 474.565 | 2.6437 | 93.80 | 2 | 6 | 6 | 35 | Yes |
| MLN-8054 | 476.870 | 5.8680 | 87.47 | 2 | 5 | 4 | 34 | No |
| KPT-330 | 443.311 | 3.3867 | 97.62 | 2 | 7 | 5 | 31 | Yes |
| Talazoparib | 380.358 | 2.6285 | 88.49 | 2 | 6 | 2 | 28 | Yes |
| CD-437 | 398.502 | 6.3784 | 57.53 | 2 | 2 | 3 | 30 | No |
| BMS-754807 | 461.505 | 3.6051 | 116.13 | 3 | 8 | 6 | 34 | Yes |
| CCT-251545 | 421.932 | 3.9090 | 63.05 | 1 | 5 | 3 | 30 | Yes |
| 3-Cl-AHPC | 408.925 | 6.2782 | 57.53 | 2 | 2 | 4 | 29 | No |
| PF-184 | 619.097 | 3.9938 | 146.60 | 4 | 8 | 7 | 44 | No |

**Abbreviations:** MW, molecular weight; TPSA, topological polar surface area; HBD, hydrogen bond donors; HBA, hydrogen bond acceptors.
